# Glycan-encoded immune checkpoints and allorecognition: a mechanistic framework for transplantation and organ engineering

**DOI:** 10.3389/frtra.2026.1841203

**Published:** 2026-06-04

**Authors:** Md Mohosin Rana, Stephen G. Withers, Jonathan C. Choy, Jayachandran N. Kizhakkedathu

**Affiliations:** 1 Centre for Blood Research, Faculty of Medicine, University of British Columbia, Vancouver, BC, Canada; 2 Department of Pathology and Laboratory Medicine, Faculty of Medicine, University of British Columbia, Vancouver, BC, Canada; 3 Department of Chemistry, Faculty of Science, University of British Columbia, Vancouver, BC, Canada; 4 Department of Molecular Biology and Biochemistry, Faculty of Science, Simon Fraser University, Burnaby, BC, Canada; 5 The School of Biomedical Engineering, University of British Columbia, Vancouver, BC, Canada

**Keywords:** allorecognition pathways, complement factor H, C-type lectin receptors, glycan-mediated immunity, glycocalyx, NK-cell glycan recognition, siglec signaling

## Abstract

Glycans constitute a structurally diverse and immunologically instructive layer that shapes how transplanted tissues are interpreted by the host immune system. Although glycoengineering approaches and glycocalyx-focused strategies have gained momentum, the mechanistic pathways through which immune cells decode glycan information remain underexplored in transplantation biology. This hybrid Perspective integrates selected mechanistic foundations with a broader conceptual framework that positions glycans as upstream immune checkpoints governing graft recognition and early innate–adaptive integration. We synthesize advances across four major axes of glycan-regulated immunity: Siglec (Sialic acid-binding immunoglobulin-type lectin)-mediated inhibitory circuits that calibrate macrophage, neutrophil, and NK-cell activation; C-type lectin receptor pathways that program antigen-presenting cells and govern antigen routing; NK-cell glycan-sensing mechanisms shaped by sialylation density, glycan topology, and ischemia–reperfusion–induced glycocalyx collapse; and complement regulation through Factor H, which interprets sialic acid motifs to restrain alternative pathway amplification. We further examine how these innate pathways intersect with glycan-dependent modulation of direct, indirect, and semi-direct allorecognition, including effects on MHC stability, exosomal transfer, antigen uptake, and T-cell intrinsic glycan checkpoints. Together, these mechanisms reveal that glycans function as a pre-recognition code that precedes and conditions classical protein-centric checkpoints by initiating, amplifying and sustaining the classical pathways, and influencing whether grafts are classified as self-like, stressed, or foreign. By consolidating these pathways into a unified model, this Perspective highlights glycan composition and architecture as a foundational design parameter for next-generation immune-compatible organ modifications and outlines mechanistic priorities for advancing glycan-informed strategies in transplantation.

## 1 Introduction

Organ transplantation has transformed the treatment landscape for end-stage organ failure, yet durable graft tolerance remains an elusive goal. Despite major advances in immunosuppression, donor–recipient matching, and emerging approaches such as xenotransplantation and stem cell–derived organ substitutes, immune rejection persists as the central barrier to long-term graft survival (55). Most current strategies to mitigate rejection focus on protein-level immunomodulation, including pharmacological inhibition of signaling proteins, costimulatory blockade, human leukocyte antigen (HLA) gene-editing, and engineered expression of immunoregulatory molecules such as programmed death-ligand 1 (PD-L1) or CD47 (38, 52, 70). While these approaches address essential components of the adaptive immune response, they often overlook an equally foundational layer of immune recognition: the glycan-rich surface architecture of organs that shapes how innate and adaptive immune cells interpret tissue identity (3, 80).

Glycans encode structurally complex signals that immune cells continuously assess to determine whether a tissue is stressed or foreign. Variations in sialylation, fucosylation, branching, and spatial presentation influence the activity of macrophages, dendritic cells, natural killer (NK) cells, and complement components (39). Importantly, glycan-guided recognition occurs upstream of many protein-based checkpoints, serving as a biochemical filter that determines whether immune responses proceed toward tolerance, inflammation, or cytotoxicity (35, 69). Yet much of the transplant-focused literature, including recent reviews on the glycocalyx, has concentrated on glycoengineering technologies or biomaterial reconstruction, leaving unresolved the key question of how immune cells mechanistically interpret glycan information during early host–graft encounters (6, 82).

A critical aspect of this mechanistic landscape involves, for example, involves the complement system, which integrates tightly with glycan biology. Complement regulators such as Factor H (FH) recognize sialic acid–rich and polyanionic glycan motifs to suppress amplification of the alternative pathway (9). This enables healthy host tissues to recruit complement inhibitors and maintain quiescence. In contrast, graft tissues that undergo ischemia–reperfusion injury (IRI) can exhibit altered sialylation and/or loss of polyanionic signatures that reduce FH binding and weaken local complement control (99). The result is unchecked C3 convertase formation, amplification, rapid complement deposition, and early inflammatory injury. Complement thus constitutes a glycan-dependent innate immune checkpoint, analogous to Siglec-mediated inhibition and the NK cell- sialic acid sensing axis and plays a decisive role in determining whether a graft is perceived as protected host tissue or as a target for immune cell binding/activation.

**Figure 1:**
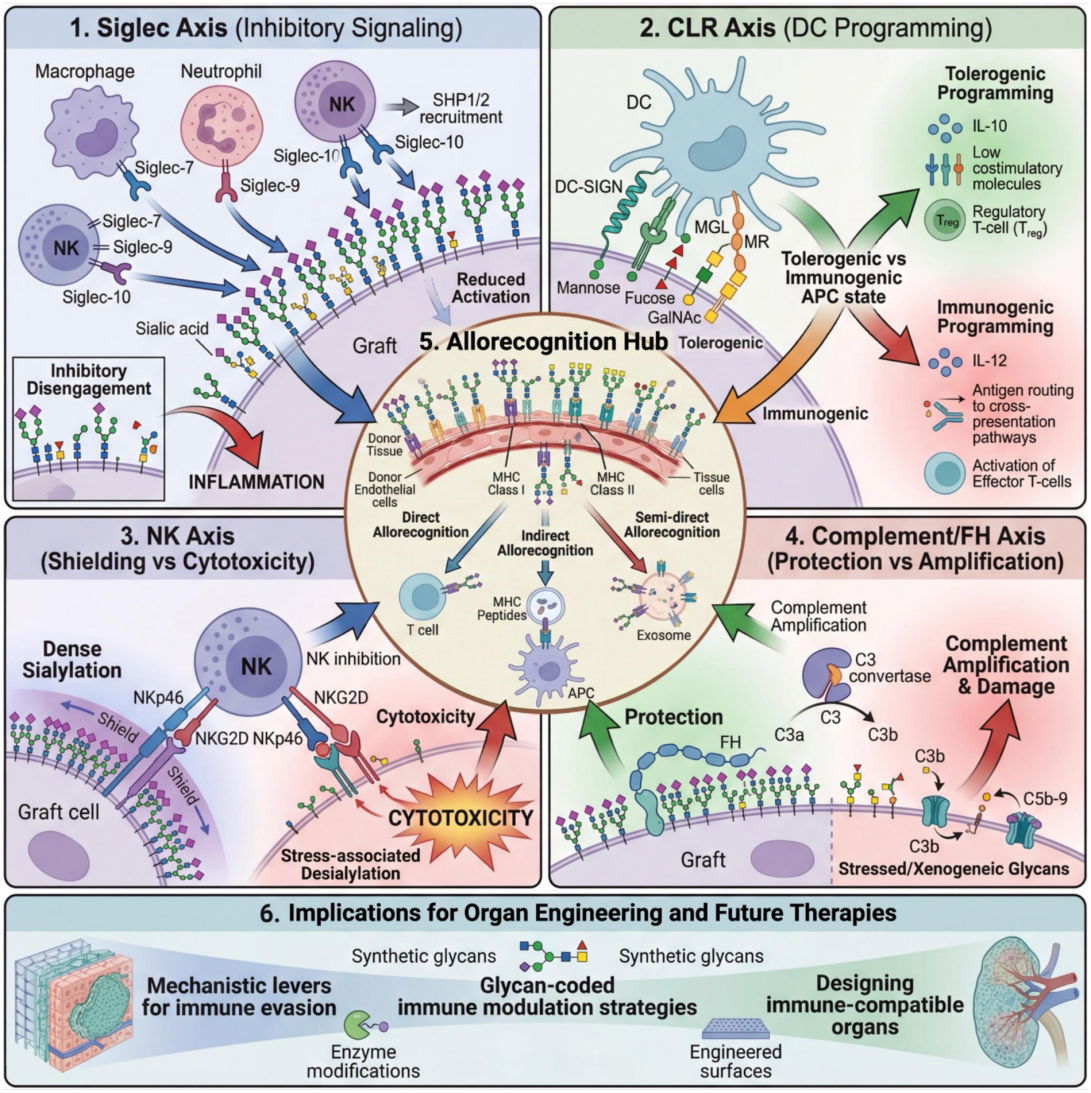
Glycan-governed immune pathways shaping host–graft interactions. This schematic integrates four major glycan-regulated axes that determine early immune interpretation of transplanted tissues. **(1)** Siglec Axis: Sialylated glycans engage inhibitory Siglecs on macrophages, neutrophils, and NK cells to dampen activation, whereas glycan loss disengages inhibition and promotes inflammation. **(2)** CLR Axis: C-type lectin receptors (DC-SIGN, MGL, MR) sense mannose-, fucose-, and GalNAc-containing glycans to program dendritic cell states, directing tolerogenic versus immunogenic antigen routing. **(3)** NK Axis: NK-cell activity reflects the balance between inhibitory signaling from dense sialylation and activation triggered by stress-associated desialylation, regulating NKG2D and NKp46 responses. **(4)** Complement/Factor H Axis: Factor H binds sialylated motifs to restrain alternative pathway activation; stressed or xenogeneic glycans reduce FH recruitment, enabling complement amplification and damage. **(5)** Allorecognition Hub: These glycan cues converge on direct, indirect, and semi-direct allorecognition by influencing MHC stability, antigen handling, and exosomal transfer. **(6)** Implications: Together, these axes highlight glycan architecture as a design parameter for engineering immune-compatible organ modifications and developing glycan-informed immunomodulatory therapies.

This manuscript is presented as a hybrid Perspective, using mechanistic background to support a broader conceptual framework on glycan-regulated transplant immunity. This Perspective therefore shifts the focus from engineering tools to the immune mechanisms by which glycans modulate graft interpretation across innate and adaptive pathways. We highlight the role of Siglec-controlled inhibitory circuits and lectin–glycan signaling on dendritic cells and macrophages, NK-cell recognition shaped by sialylation and glycan topology, and glycan-dependent complement checkpoints including FH and alternative pathway amplification (Figure 1). We further integrate emerging insights into T-cell-intrinsic glycan checkpoints, Fc-glycan regulation of antibody effector function, and the impact of ischemia–reperfusion–driven glycocalyx collapse/shedding on early innate activation. Finally, we synthesize how these glycan-mediated cues influence direct, indirect, and semi-direct allorecognition pathways, altering major histocompatibility complex (MHC) stability, antigen routing, exosomal transfer, and related non-self-glycan incompatibilities. Rather than cataloging engineering techniques, our goal is to clarify how immune cells read the glycan code and how this mechanistic knowledge can guide rational organ modification design.

Because transplantation-specific evidence varies across these pathways, we explicitly distinguish mechanisms supported in transplant or ischemia–reperfusion settings from those inferred from cancer, infection, vascular biology, xenotransplantation, or basic immunology. Accordingly, the glycan-checkpoint model is framed as an integrative mechanistic framework, with indirectly supported mechanisms identified as transplant-relevant hypotheses requiring validation in allograft or xenograft settings.

### 1.1 Key terminology

In this Perspective, we use “glycan checkpoint” to describe a glycan-dependent regulatory mechanism that alters immune activation thresholds, such as Siglec-mediated inhibition, CLR-directed APC programming, NK-cell ligand masking, or Factor H recruitment. We use “glycan code” to refer to the combinatorial immune information encoded by glycan composition, linkage, branching, density, accessibility, and spatial organization. We use “inhibitory tone” to denote the net suppressive signaling imposed by inhibitory glycan-binding pathways, particularly Siglec-dependent signaling, as determined by ligand abundance, receptor engagement, glycan accessibility, and microdomain organization.

To clarify how strongly each pathway shown in Figure 1 is supported by transplantation-specific evidence, Table 1 summarizes the relative evidentiary strength for the major glycan-regulated mechanisms discussed in this Perspective.

**Table 1:** Relative evidentiary strength of glycan-regulated pathways in transplantation.

Glycan-regulated pathway	Evidence strength in transplantation	Framing in this Perspective	Representative evidence
IRI-induced endothelial glycocalyx collapse/shedding	Strong	Established upstream injury mechanism linking reperfusion injury to innate activation and graft immunogenicity	(1, 25, 82)
Bioengineering/glycocalyx rebuild	Moderate to strong	Emerging interventional strategy that moves glycan biology from descriptive injury mechanism to graft-surface engineering; strongest evidence comes from vascular allograft glycocalyx protection and emerging endothelial glycocalyx-mimetic polymer approaches	(62, 93)
Factor H/Complement glycan regulation	Strong	Established glycan-sensitive checkpoint controlling alternative pathway amplification and endothelial injury	(9, 58, 60)
Glycan removal or modification in ABO-incompatible transplantation	Strong	Clinically established ABO glycan barrier with emerging donor-organ engineering strategies to enzymatically remove or convert A/B antigens and reduce anti-ABO antibody binding, complement activation, and hyperacute injury.	(65, 74, 106, 110, 118, 119)
Xenogeneic glycan antigens	Strong	Established glycan barrier in xenotransplantation through natural antibody binding and complement activation	(11, 59, 98)
Siglec-mediated inhibitory signaling	Moderate	Transplant-relevant inhibitory checkpoint; strongest evidence from murine transplant models and human biopsy associations	(10, 87)
CLR-mediated APC programming	Indirect to moderate	Plausible modifier of antigen uptake, APC maturation, and indirect allorecognition	(8, 89, 101)
NK-cell glycan sensing	Indirect	Reperfusion-phase mechanism that may modulate NK activation thresholds alongside MHC-dependent regulation	(21, 25, 122)
Direct allorecognition via MHC glycosylation	Limited	Context-dependent modifier of peptide-MHC stability and TCR engagement, not a replacement for HLA mismatch	(5, 31, 84)
Indirect allorecognition via glycan-dependent uptake	Indirect to moderate	Plausible pathway linking injury-altered donor glycans to recipient APC antigen processing	(44, 63)
Semi-direct allorecognition via extracellular vesicle glycans	Limited	Testable hypothesis linking donor EV glycosylation to APC targeting and cross-dressing efficiency	(37, 85, 112)
T-cell intrinsic glycan checkpoints	Limited	Potential recipient-side modifier of alloreactive T-cell activation thresholds	(22, 24, 40)
B-cell and antibody Fc-glycan regulation	Moderate to strong	Transplant-relevant modifier of donor-specific antibody effector function and antibody-mediated injury	(12, 18, 107)

## 2 Siglec-mediated inhibitory pathways: glycan-mapped immune checkpoints

### 2.1 Overview of the siglec family in innate immune regulation

**Figure 2:**
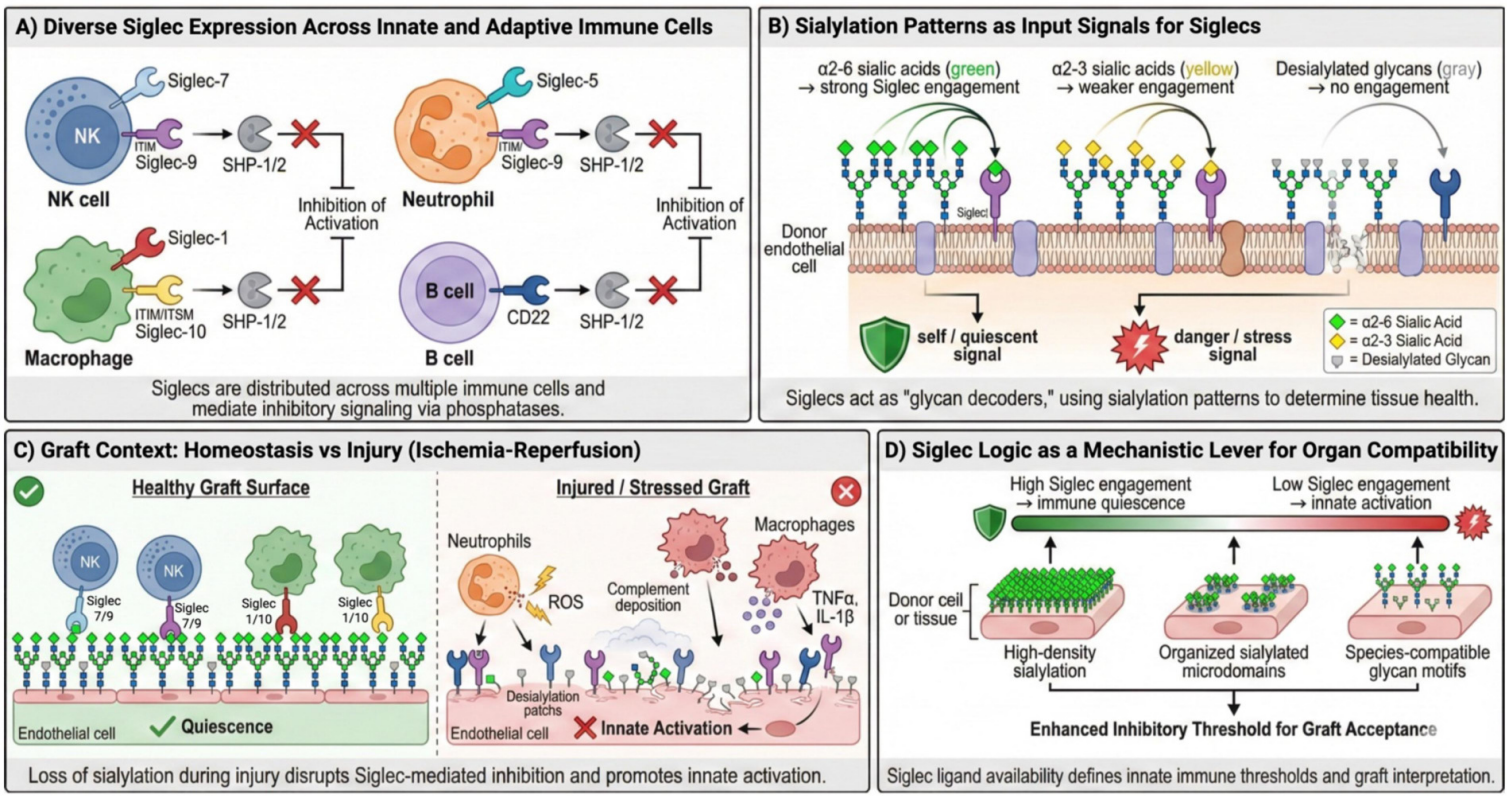
Siglec-mediated glycan sensing as a regulator of graft interpretation. **(A)** Innate and adaptive immune cells express distinct inhibitory Siglecs that recruit SHP-1/2 to suppress activation. **(B)** Siglecs decode graft sialylation patterns: *α*2–6 sialic acids strongly engage inhibitory receptors and signal tissue quiescence, *α*2–3 linkages give weaker engagement, and desialylated glycans fail to engage Siglecs and convey stress cues. **(C)** Healthy grafts maintain dense sialylation to support Siglec-mediated quiescence, whereas ischemia–reperfusion injury reduces sialylation, exposing desialylated patches that promote complement deposition and innate activation. **(D)** Graft sialylation density, microdomain organization, and species-compatible glycan motifs collectively set the level of Siglec engagement and the inhibitory threshold for graft acceptance.

Sialic acid–binding immunoglobulin-like lectins (Siglecs) constitute a central class of glycan-sensing receptors that translate cell-surface glycan patterns into immunoregulatory signals (56). Expressed across multiple immune subsets, including macrophages, neutrophils, dendritic cells, NK cells, some types of T-cells, monocytes, and B cells, Siglecs function as inhibitory checkpoints that help maintain immune homeostasis and prevent inappropriate activation against healthy tissues (56, 87). Many Siglecs contain ITIM (immunoreceptor tyrosine-based inhibitory motif) or ITSM (switch motif) domains in their cytoplasmic tails. Upon ligand engagement, these motifs recruit Src homology region 2 domain-containing phosphatase SHP-1 and, in some contexts, SHP-2. SHP-1 serves as a canonical negative regulator of immune receptor signaling, whereas SHP-2 can play more context-dependent roles but also contributes to inhibitory signaling downstream of Siglec engagement. Their recruitment attenuates proximal signaling pathways such as spleen tyrosine kinase (SYK), zeta-chain-associated protein kinase 70 (ZAP-70), and nuclear factor-kappa B (NF-*κ*B) activation (Figure 2A). This results in reduced phagocytic activity, limited cytokine release, and restrained cytotoxic function.

Although Siglecs are broadly conserved as sialic-acid–binding inhibitory receptors, the Siglec repertoire differs substantially between humans and mice, particularly within the rapidly evolving CD33-related Siglec subgroup, making strict one-to-one orthology and functional extrapolation imperfect (13). Several Siglecs commonly emphasized in human innate checkpoint regulation (e.g., Siglec-7 and Siglec-9) lack direct murine orthologs; conversely, mice express related receptors (e.g., Siglec-E) that are often used as functional analogs for selected pathways (42). Accordingly, unless otherwise specified, this Perspective focuses on human Siglec biology and references murine Siglecs only where they serve as established experimental counterparts.

While structurally related, individual Siglecs exhibit unique ligand-binding preferences and immune roles. CD22 (Siglec-2) on B cells modulates BCR (B-cell receptor) signaling and supports tolerance by engaging *α*2–6 sialylated ligands (71). In humans, Siglec-7 and Siglec-9, expressed predominantly on NK cells, effector T cells, neutrophils, and subsets of monocytes, sense sialylated patterns on self-cells to limit activation, degranulation, and inflammatory responses (67). Siglec-10, expressed on human macrophages and some dendritic cell subsets, participates in “do-not-eat-me” signaling, binding CD24 and other sialylated ligands to restrain phagocytosis in sterile inflammation (7). However, the molecular basis, generality, and context dependence of the Siglec-10-CD24 interaction have recently been questioned, indicating that this axis should be interpreted cautiously and requires further validation in transplantation-relevant settings (94). Lesser-studied Siglecs, including Siglec-5, -8, -14, and −15, further contribute to innate immune calibration by recognizing context-dependent glycan motifs and modulating inflammatory tone (56, 67). Together, these receptors form a distributed network of glycan-based inhibitory checkpoints, allowing immune cells to use sialic-acid signals as a proxy for tissue health, self-identity, or cellular stress.

### 2.2 Sialylation patterns as immunological “input signals”

The ability of Siglecs to act as inhibitory regulators depends on the specificity and presentation of sialylated glycans on surrounding tissues. Here, we use “self” to denote recipient-compatible, homeostatic sialoglycan patterns that sustain tonic engagement of inhibitory receptors (including Siglecs) and thereby impose an inhibitory baseline on innate cells. This is distinct from “species-specific” glycans, which reflect interspecies differences in glycosylation pathways and may introduce epitopes absent from the human sialome. Immune cells discriminate among *α*2–3, *α*2–6, and *α*2–8 linkages, which can bias lectin engagement and downstream signaling in a context- and presentation-dependent manner (Figure 2B). For instance, *α*2–6 sialylation is enriched on healthy epithelial and endothelial surfaces and is particularly effective in engaging inhibitory Siglecs such as CD22 and Siglec-10, and can also interact with other inhibitory Siglecs including Siglec-7 and Siglec-9 depending on glycan density, presentation, and underlying glycan structure (103). In contrast, *α*2–3 linkages are often associated with dynamic or stressed cellular states, including injury, inflammation, and pathogen exposure (123). *α*2–8 Disialic structures, found on neural tissue and some immune cells, may convey highly specialized contextual information (104). Although both Siglec expression and sialoglycan ligands vary across individuals and cell states, current evidence supports a largely graded, quantitative “inhibitory tone” (e.g., density/architecture and accessibility of sialylated motifs) rather than a discrete, KIR-like haplotype system that categorically partitions human populations for Siglec-mediated recognition.

Under homeostatic conditions, tissues maintain a high baseline sialylation density, enabling persistent engagement of inhibitory Siglecs and supporting immune quiescence. This “sialylation shield” serves as a molecular signature of healthy self-tissue. However, during injury, hypoxia, metabolic stress, or enzymatic remodeling, sialylation can become significantly altered (120, 123). Ischemia–reperfusion injury or IRI, a common event during organ procurement and transplantation, leads to glycan degradation, reduced sialyltransferase activity, and exposure of desialylated glycans (25). These changes are perceived by Siglec-bearing immune cells as danger-associated molecular patterns, resulting in reduced inhibitory signaling and increased immune activation.

Importantly, innate recognition of allogeneic non-self is not limited to Siglec pathways. Seminal work over the past 10–20 years has demonstrated that monocytes/macrophages can exhibit forms of allorecognition and “innate allomemory,” including via mismatched inhibitory axes such as CD47–SIRP*α* and other MHC-I–sensing systems (20, 78, 121). While these mechanisms are not strictly glycan-mediated, they reinforce a common principle central to this Perspective: early myeloid activation thresholds are shaped by “don't-attack-me” baselines, and disruption of these baselines, whether through receptor–ligand mismatch or injury-driven glycocalyx remodeling, can condition antigen presentation and amplify downstream adaptive alloresponses.

Thus, sialylation patterns act as immunological input signals: dense, structurally organized and intact sialylation promotes tolerance, whereas altered or reduced sialylation lowers activation thresholds and primes innate immune cells for effector responses.

### 2.3 Role in transplant immunobiology

Given their sensitivity to glycan context, Siglecs play a crucial role in shaping the early inflammatory landscape following organ transplantation. Innate immune cells infiltrating the graft rapidly assess sialic-acid density (related to multivalency), linkage preferences, and spatial organization on donor endothelium and parenchyma. Even modest changes in these parameters can dramatically shift the immune set-point. Sialylation density influences the strength and stability of Siglec engagement. High-density sialylation promotes robust inhibitory signaling, suppressing neutrophil activation and limiting macrophage inflammatory responses. Conversely, patchy or reduced sialylation, which often accompanies ischemic injury, reduces Siglec engagement that is related to greater accumulation of activating signals such as damage-associated molecular patterns (DAMPs) and unmasked glycan motifs. This could tip the balance toward inflammation and early graft damage (Figure 2C) (72). Consistent with this framework, recent work from Fairchild and colleagues identified Siglec-E as an innate immune checkpoint in murine heart transplantation: recipient Siglec-E restrained dendritic-cell activation, NF-*κ*B signaling, TNF-α production, and downstream alloreactive T cell priming, whereas Siglec-E deficiency accelerated acute rejection. The same study further showed that the human paralogs Siglec-7 and Siglec-9 were upregulated in biopsies taken from rejecting allografts, and higher expression correlated with improved allograft survival, supporting the translational relevance of this inhibitory Siglec axis in transplant immunobiology (10).

Linkage patterns further modulate these outcomes. Grafts enriched in *α*2–6 sialylation engage inhibitory Siglecs more effectively than those dominated by *α*2–3 linkages, a difference that may help explain the variable innate reactivity observed across tissue types and species. This is particularly relevant in xenotransplantation, where discordant glycan structures, such as distinct sialylation motifs or non-human sialic acids, may fail to engage human Siglecs, resulting in heightened NK cell activity, macrophage activation, and complement-mediated injury.

Spatial distribution of sialylation also matters. Organized microdomains of sialylated glycoconjugates on healthy endothelia promote stable Siglec clustering and inhibitory signaling (Figure 2D). In contrast, disorganized or fragmented glycan landscapes, typical of stressed or damaged grafts, disrupt Siglec engagement and contribute to immune activation. These effects complement the activation seen upon IRI, where rapid glycan remodeling and desialylation and glycocalyx shedding create a window of heightened innate vulnerability.

Together, these mechanisms highlight Siglec pathways as critical determinants of innate immune reactivity during transplantation, influencing macrophage recruitment, neutrophil priming, NK cell cytotoxicity, and even early complement activation through altered surface homeostasis.

### 2.4 Implications for organ bioengineering

The mechanistic insights described above highlight an important conceptual point: modulating Siglec ligands equates to altering innate immune set-points and targets the earliest events that define alloimmune activation.

For organ bioengineering, this suggests that graft tolerance may not be achieved solely through protein-level immunomodulation. The glycan landscape must be designed with Siglec biology in mind to ensure that immune cells interpret the graft as “self-like” from the earliest moments of contact. While this Perspective does not focus on specific engineering strategies, the mechanistic principle is clear: a graft enriched in appropriate sialylation patterns is inherently more likely to engage inhibitory Siglecs, dampen innate activation, and promote immune quiescence. Conversely, a graft presenting altered or noncanonical glycans may unintentionally lower Siglec-mediated inhibition and trigger inflammatory pathways, even in the absence of classical alloantigen mismatches (Figure 2D).

Future organ engineering design must therefore consider endogenous Siglec pathways as tunable immunological levers. By maintaining or reconstructing glycan contexts that promote Siglec engagement, bioengineered tissues may achieve a more stable and predictable immune profile, reduce early innate aggression and improve downstream adaptive outcomes. Understanding the mechanistic underpinnings of Siglec signaling thus provides a foundation for integrating glycan-mediated immune checkpoints into next-generation transplantation engineering.

## 3 Lectin-mediated activation and reprogramming of APCs

### 3.1 C-type lectin receptors (CLR) biology overview

**Figure 3:**
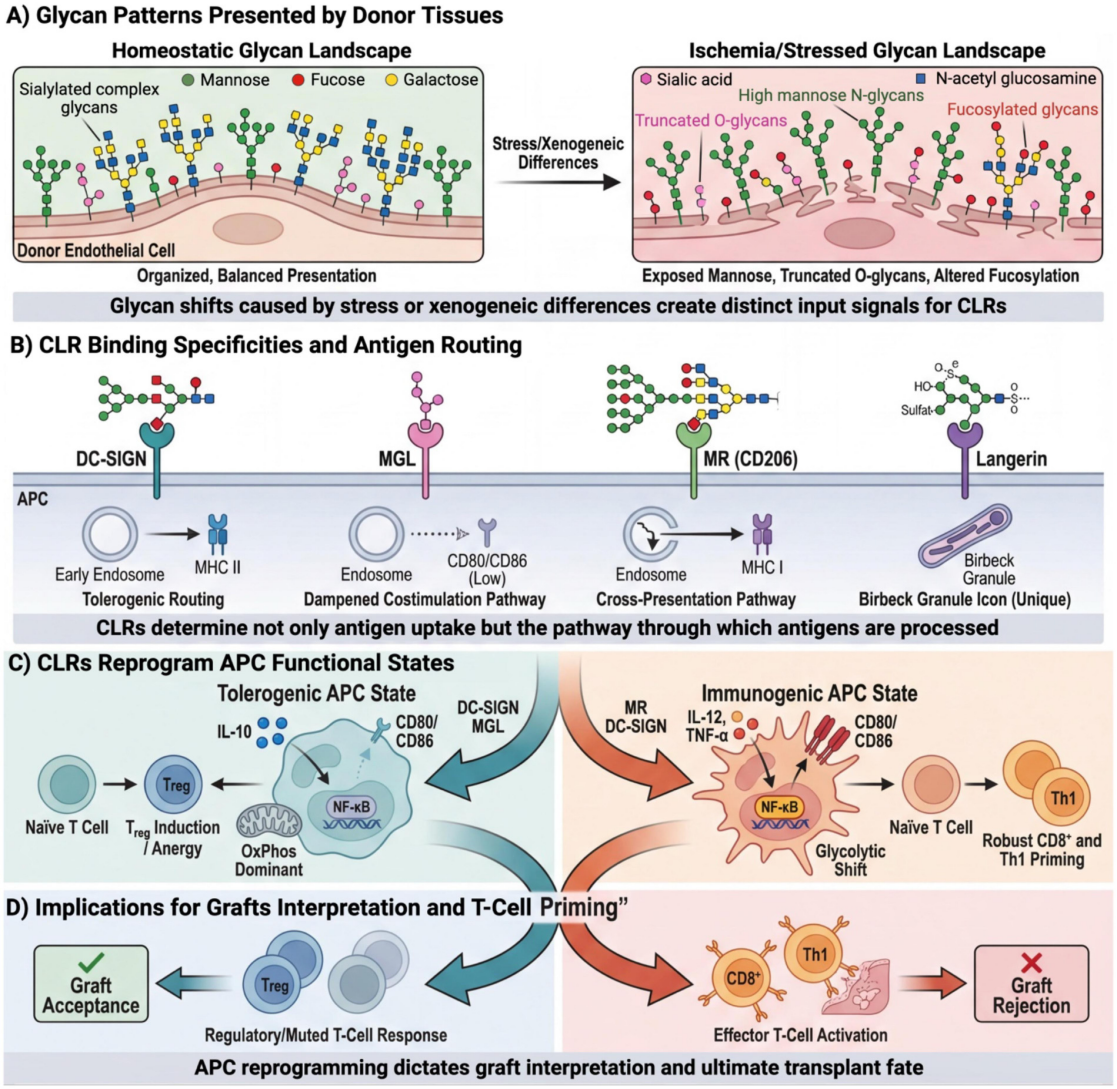
CLR sensing of donor glycan patterns drives APC programming and shapes graft immunity. **(A)** Homeostatic vs. stressed/xenogeneic donor tissues display distinct glycan landscapes that generate different inputs for C-type lectin receptors (CLRs). **(B)** CLRs such as DC-SIGN, MGL, MR, and Langerin bind specific glycan motifs and route antigens into tolerogenic or immunogenic pathways. **(C)** These signals reprogram APC states, promoting IL-10–rich, low-costimulation tolerogenic profiles or IL-12/TNF-α–driven immunogenic profiles. **(D)** Resulting T-cell priming favors either regulatory responses that support graft acceptance or effector CD8⁺/Th1 responses that drive rejection.

C-type lectin receptors (CLRs) comprise a diverse family of glycan-binding pattern recognition receptors prominently expressed on dendritic cells (DCs), macrophages, Langerhans cells, and some monocytes (83). Unlike Siglecs, which detect sialylated “self-associated molecular patterns”, CLRs recognize a broader repertoire of glycan motifs that are altered or absent when homeostasis is disrupted, enabling them to detect altered tissue states, pathogens, and environmental cues (Figure 3A). Among the most relevant CLRs for transplant immunobiology are DC-SIGN (dendritic cell-specific ICAM-3 grabbing non-integrin, CD209), MGL (macrophage galactose-type lectin), MR (mannose receptor, CD206), and Langerin (CD207).

Each of these molecules recognizes a distinct set of glycan patterns: DC-SIGN binds mannose-rich and fucosylated structures, including high-mannose glycans and Lewis-type antigens (26). MGL selectively engages N-acetylgalactosamine (GalNAc) residues, often found on truncated O-glycans or stress-associated glycoforms (116). MR, with its multiple carbohydrate recognition domains, recognizes mannose, fucose, and N-acetylglucosamine (GlcNAc), facilitating broad antigen scavenging (19). Langerin binds sulfated and mannose-rich glycans and directs them to specialized Birbeck granules for antigen processing (Figure 3B) (97).

CLR engagement governs multiple fundamental aspects of antigen presentation and APC programming. First, CLRs mediate antigen uptake, often with greater affinity for glycosylated antigens than for peptide-based ligands. Second, they control endosomal routing, determining whether internalized material enters degradative pathways, recycling compartments, or cross-presentation channels. Third, CLR signaling modulates DC maturation, influencing expression of MHC molecules, costimulatory ligands (CD80/CD86), and cytokines such as IL-10 and IL-12. Through these combined activities of glycan recognition, antigen routing, and signal transduction, CLRs act as key regulators of APC activation that define how the adaptive immune system responds to antigen exposures (16, 66).

### 3.2 Mechanistic role in tolerogenic vs. immunogenic APC states

CLR signaling can drive APCs toward tolerogenic or immunogenic states depending on the glycan structures encountered, receptor-specific motifs, and cellular context (Figure 3C).

#### 3.2.1 Tolerogenic programming via DC-SIGN and MGL

Engagement of DC-SIGN by certain fucosylated ligands stimulates pathways that enhance IL-10 secretion and reinforce a regulatory phenotype. These DC-SIGN–mediated signals inhibit NF-*κ*B activation, promote signal transducer and activator of transcription 3 (STAT3)-dependent anti-inflammatory programs, and attenuate DC maturation, yielding APCs that encourage peripheral tolerance rather than effector T-cell activation (Figures 3C,D) (14, 49). Similarly, MGL engagement by GalNAc-containing glycans has been associated with tolerogenic functions. MGL ligation can modulate intracellular signaling cascades leading to reduced expression of costimulatory molecules and increased presentation of antigens within non-inflammatory pathways. Because MGL often recognizes truncated or stress-associated O-glycans, its activation may serve as a checkpoint preventing excessive inflammation in settings where cells exhibit altered glycosylation but are not overtly pathogenic (102, 117).

#### 3.2.2 Immunogenic activation via mannose-rich ligands

Conversely, recognition of mannose-rich structures by CLRs such as MR or DC-SIGN can promote immunogenic programming, particularly when these structures resemble pathogen-associated molecular patterns (2). MR-mediated internalization frequently delivers antigens into cross-presentation pathways, enhancing MHC I loading and priming CD8⁺ T-cell responses (57). These mannose-driven interactions can also increase expression of costimulatory receptors and inflammatory mediators, tilting APCs toward an immunogenic profile.

#### 3.2.3 Metabolic and cytokine reprogramming

CLR engagement exerts additional control by shaping the APC metabolic state, which is tightly linked to functional outcomes. Tolerogenic signaling often promotes oxidative phosphorylation and lipid metabolism, whereas immunogenic activation shifts cells toward glycolysis. Distinct CLR ligands also modulate cytokine programs: IL-10, transforming growth factor-*β* (TGF-*β*), and retinoic acid pathways are associated with tolerogenic responses, while interleukin-12 (IL-12), tumor necrosis factor-*α* (TNF-α), and type I interferons (IFNs) characterize immunogenic activation (Figures 3C,D) (66, 91).

Thus, CLRs function not merely as endocytic receptors but as glycan-sensing switches that determine the immunological fate of antigens and influence DC–T-cell crosstalk.

### 3.3 Relevance to transplantation and engineered organs

The nature of these glycans, species-specific motifs, injury-induced alterations, and stress-associated patterns, directly influences whether recipient APCs interpret the graft as self-like, “altered self,” or “pathogen-like.”

#### 3.3.1 Donor glycans as determinants of APC activation

Healthy tissues typically present organized, homeostatic glycan patterns, including balanced fucosylation and limited exposure of high-mannose structures. These patterns engage CLRs in ways that favor controlled antigen uptake and non-inflammatory routing. However, during procurement and ischemia–reperfusion, donor tissues undergo significant remodeling: mannose-rich glycans may become more exposed, O-glycan truncation may occur, and fucose presentation may shift (82). These changes are interpreted by CLRs as danger-associated signals, promoting immunogenic activation.

#### 3.3.2 IRI-mediated glycocalyx injury

Ischemia–reperfusion rapidly disrupts the endothelial glycocalyx through oxidative stress, shear stress changes, and protease/heparinase activity, driving shedding of core proteoglycans (e.g., syndecan-1) and release of glycosaminoglycans (e.g., heparan sulfate) into the perfusate/circulation. Glycocalyx loss exposes the denuded endothelial surface to leukocyte adhesion and amplifies complement and cytokine cascades that typify early reperfusion injury, including increased vascular permeability and myeloid recruitment. In kidney transplantation, endothelial glycocalyx injury has been highlighted as a key upstream event linking IRI to delayed graft function (DGF) and heightened immunogenicity of the graft microvasculature (1, 25).

Concurrently, IRI alters the glycan landscape presented to innate lectins by (i) degrading terminal “self-associated” features (notably sialylation and polyanionic glycans) and (ii) increasing the relative display or accessibility of mannose-/fucose-rich motifs on damaged endothelium and extracellular debris, consistent with injury-driven shifts in glycan processing rather than *de novo* synthesis alone. These changes create a favorable context for C-type lectin receptor (CLR) engagement. For example, the macrophage mannose receptor (MR/CD206) is an endocytic lectin that scavenges endogenous mannosylated ligands and tissue-damage–derived glycoprotein fragments, promoting antigen uptake and routing into processing pathways that can increase antigen availability for presentation (26, 101). In parallel, DC-SIGN/CD209 family receptors bind high-mannose and fucosylated glycans and can couple ligand capture to signaling programs that shape inflammatory cytokine output and DC maturation, thereby influencing the quality and magnitude of subsequent T-cell priming (89). Consistent with this concept, multiple transplant/IRI models demonstrate that IRI increases myeloid DC maturation/trafficking and enhances cross-presentation capacity, mechanistically aligning glycocalyx collapse/shedding and CLR-driven uptake with accelerated development of adaptive allo-immunity (8, 63).

#### 3.3.3 Shaping lymph node priming

Once CLR-conditioned APCs migrate to draining lymph nodes, their glycan-mediated programming directly influences T-cell fate. Tolerogenic APCs may induce regulatory T cells or anergy, while immunogenic APCs promote robust effector differentiation (81). Thus, glycan-determined APC trajectories represent a central decision point in transplant immunobiology.

Although CLR-mediated antigen uptake and APC programming are well established in dendritic cell biology, direct causal evidence linking specific donor glycan motifs to indirect allorecognition in transplantation remains limited. However, ABO-incompatible transplantation provides an important example in which carbohydrate antigens clearly shape graft-directed immunity and may also give rise to accommodation, a state in which the graft resists antibody- and complement-mediated injury despite persistent or returning anti-ABO antibodies (28). We therefore frame CLR–glycan interactions as a transplant-relevant but incompletely validated mechanism, while recognizing ABO accommodation as stronger clinical evidence that glycan-directed immune responses can follow trajectories distinct from protein alloantigen responses.

#### 3.3.4 Xenogeneic glycan signatures as a special case of glycan-encoded checkpoints

Xenografts introduce additional complexity. Species-specific glycan structures, such as distinct fucosylation patterns, unique branching motifs, or modified GalNAc structures, may be recognized by human CLRs as pathogen-like (53, 96). Such recognition can accelerate immunogenic DC maturation, increase antigen cross-presentation, and enhance T-cell responses, thereby amplifying xenograft rejection risk even in the absence of classical antigen mismatches.

### 3.4 Mechanistic implications for organ design

The mechanistic insights outlined above emphasize that CLR–glycan interactions define APC functional outcomes, independent of any engineered organ modifications. This has significant implications for organ design: controlling graft glycan patterns conceptually equates to influencing how recipient APCs will process and present donor antigens. By maintaining or restoring glycan contexts that favor DC-SIGN– and MGL-mediated tolerogenic cues, engineered tissues may bias early APC interpretation toward immune quiescence. Conversely, unintentional exposure of mannose-rich or stress-associated glycans could drive immunogenic activation even when protein-level antigen mismatches are minimized.

Future organ engineering design will therefore require careful consideration of lectin-driven APC programming, recognizing that CLRs are not passive endocytic receptors but central determinants of whether graft antigens are framed as benign or immunogenic. Understanding these mechanistic levers will be essential for integrating glycan biology into emerging frameworks for transplant tolerance and the next generation of immune-compatible engineered organs.

## 4 Glycan recognition circuits controlling NK and memory T cells

### 4.1 NK activating vs. inhibitory signals governed by glycans

Natural killer (NK) cells integrate multiple activating and inhibitory inputs to determine whether a target cell is eliminated or tolerated. Although much attention has focused on MHC class I–dependent inhibitory receptors [e.g., killer immunoglobulin-like receptors (KIRs), natural killer group 2 member A (NKG2A)], the glycan landscape on target tissues plays an equally fundamental role in shaping NK-cell decision-making (68). Glycan-mediated regulation occurs through two main mechanisms: (1) masking or exposing activating ligands, and (2) engaging sialic acid–dependent inhibitory pathways, especially via Siglecs.

**Figure 4:**
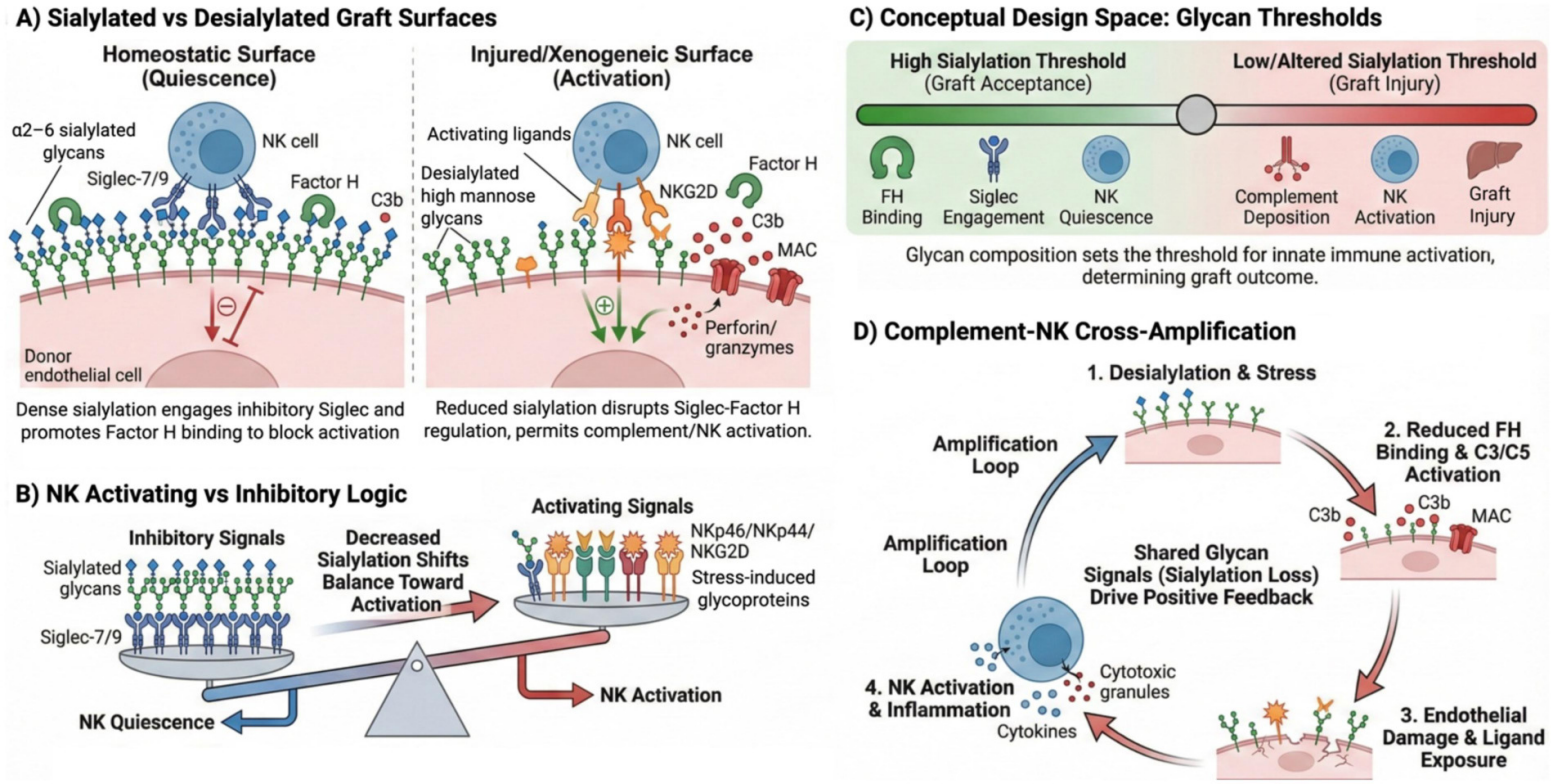
Sialylation-dependent regulation of NK-cell activation and complement control at the graft interface. **(A)** Homeostatic graft surfaces maintain dense sialylation that engages inhibitory Siglecs and recruits Factor H, suppressing NK activation and complement deposition. Injured or xenogeneic surfaces lose sialylation, expose mannose-rich glycans, reduce Factor H binding, and promote complement activation and NK cytotoxicity. **(B)** NK-cell behavior reflects the balance between inhibitory signals from sialylated glycans and activating ligands revealed by stress-associated desialylation. **(C)** Conceptual “glycan threshold” model illustrating how sialylation density sets the inhibitory tone for graft acceptance, whereas low or altered sialylation shifts toward complement deposition, NK activation, and graft injury. **(D)** Desialylation initiates a positive feedback loop: reduced Factor H binding allows C3/C5 activation, complement deposition increases endothelial injury, and ligand exposure further amplifies NK activation.

Many activating NK receptors, including NKp46, NKp44, and NKG2D, recognize ligands whose accessibility depends on glycan density and structure (68). High levels of sialylation on the target cell surface can sterically conceal these activating ligands, suppressing NK-cell engagement even when stress signals are present. In contrast, desialylation, caused by cellular stress, hypoxia, or enzymatic activity, exposes underlying glycoproteins and glycolipids, rendering them accessible to NK-activating receptors. This creates a glycan-based system of “visibility”: sialylated cells appear quiescent, whereas desialylated or damaged cells reveal activating epitopes (Figures 4A,B) (76, 103, 111).

In parallel, NK cells express Siglec-7 and Siglec-9, ITIM-containing receptors that bind sialic acid–rich ligands. Engagement of these Siglecs recruits SHP-1/2 phosphatases, suppressing activation pathways and limiting NK degranulation and cytokine production (122). Thus, sialylated glycans provide NK cells with a self-associated inhibitory signal, reinforcing tolerance toward healthy tissues. When sialic acids are lost or altered, Siglec engagement weakens, lowering inhibitory thresholds and enabling activating signals to dominate (Figure 4B).

Effector and effector-memory T cells can also display “innate-like” behavior that makes them responsive to the same glycan-coded tissue cues that calibrate NK responses (4, 48). In inflammatory or ischemic settings, highly differentiated cytotoxic T-cell populations can execute rapid cytokine release and cytolytic programs with less dependence on *de novo* priming, while integrating stress-associated signals through NK-linked pathways (e.g., NKG2D and related receptor systems) (33). Importantly, subsets of effector and memory T cells express inhibitory Siglecs (including Siglec-7 and Siglec-9 in humans), providing a mechanism by which sialic acid–rich “self-associated” glycans can raise activation thresholds and restrain effector function (36). Consequently, glycocalyx disruption and desialylation at the graft interface can simultaneously increase activating-ligand accessibility and reduce sialic acid–dependent inhibitory tone, enabling NK cells and rapidly responsive T cell subsets to function as early cytotoxic sensors of tissue injury and graft “non-selfness.”

Together, these mechanisms position NK and memory T cells as highly sensitive interpreters of the glycan landscape, rapidly translating subtle changes in sialylation, glycan branching, and ligand accessibility into actionable immune decisions.

### 4.2 Glycan topology and density

A key feature of NK-cell glycan sensing is that NK responses are not determined solely by what glycans are present, but by how they are arranged or organized on cell surface. Glycan topology, referring to density, microdomain organization, and nanoscale spacing, critically influences NK-cell activation at the immune synapse.

#### 4.2.1 Microdomain distribution and ligand clustering

Sialylated glycoconjugates often form microdomains or patches on the cell surface. These microdomains can cluster Siglec ligands and promote stable inhibitory signaling, strengthening NK tolerance. Conversely, heterogeneous or disrupted distribution, such as occurs during stress or ischemia, results in patchy inhibitory landscapes, which reduce Siglec engagement and increase exposure of activating ligands. Importantly, NK cells appear sensitive not only to whether ligands are clustered, but to how they are patterned within the synapse: nanoscale ligand spacing, local glycan density, and receptor microclustering can govern receptor stabilization, force transmission across the interface, and the probability of productive activating signaling. In this view, loss of microdomain “coherence” is read by NK cells as both a quantitative reduction in inhibitory input and a topological change in spacing/organization that lowers the activation threshold (15, 76, 103).

#### 4.2.2 Steric shielding at the immune synapse

NK-cell activation depends on the tight formation of an immune synapse. Dense glycan layers, particularly sialylated and heavily O-glycosylated mucin-like structures, can act as steric shields by increasing the effective intermembrane distance and reducing the probability that short receptor–ligand pairs (e.g., many activating interactions) can engage long enough to nucleate signaling microclusters (34). A bulky glycocalyx can also impede integrin-dependent adhesion and limit receptor diffusion/segregation, delaying the formation of a mature synapse and the downstream actin remodeling needed for degranulation. This shielding effect is not passive: disease, cellular stress, and enzymatic remodeling change glycan height (chain length/extension), branching (excluded volume), and valency (multivalency/clustering), which can (i) compress or “thin” the glycocalyx to permit closer membrane apposition, (ii) redistribute glycoproteins away from the central contact zone to create local “windows” for receptor access, and/or (iii) expose underlying protein epitopes and activating ligands by removing terminal sialic acids or truncating extended O-glycans (17). In parallel, reducing sialylation can diminish inhibitory Siglec engagement, so glycocalyx remodeling/shedding simultaneously removes a physical barrier and shifts the balance of inhibitory versus activating signaling.

### 4.3 NK and memory T cell immunobiology in the transplant setting

NK cells play a pivotal role in the early stages of graft interrogation, particularly during reperfusion and acute innate inflammation. They are among the first lymphocytes to engage the donor endothelium and are uniquely sensitive to the glycan alterations that accompany transplantation. Memory CD8T cells may also be one of the earliest immune cells recruited into heart transplants where they initiate inflammatory responses through the secretion of interferon-*γ* (88).

#### 4.3.1 NK cells in early graft injury

During procurement and reperfusion, graft tissues undergo ischemia–reperfusion injury, which disrupts sialylation, alters glycan branching, and exposes mannose-rich or truncated O-glycans (consequence of glycocalyx shedding). These changes diminish Siglec engagement and increase accessibility of NKp46, NKp44, and NKG2D ligands on NK cells and IFN*γ* secretion from memory T cells, fostering a pro-activation environment. NK cells and memory T cells recruited to the graft vasculature may thus interpret the graft as damaged or foreign, driving early inflammation, cytotoxicity, and cytokine release.

#### 4.3.2 Donor glycosylation as a regulator of NK activation thresholds

NK-cell responses in transplantation are shaped by the balance of inhibitory and activating signals encountered within the graft. Although NK-cell licensing, the process by which NK cells become functionally competent after inhibitory receptor interactions with self MHC class I during development, is established before transplantation, donor glycan patterns may still influence the activation threshold of recipient NK cells at the graft interface (115). For example, a graft with preserved, human-compatible sialylation may engage inhibitory Siglecs more effectively and thereby help restrain NK activation (21, 122). In contrast, reduced or species-incompatible sialylation, as may occur in xenotransplantation or during ischemia-reperfusion injury, may weaken these inhibitory signals and favor NK-cell cytotoxicity in the presence of concurrent activating stimuli (25, 27).

Evidence from tumor and stress-recognition models strongly supports the principle that altered sialylation can regulate NK-cell activation through ligand masking and Siglec engagement. In transplantation, this mechanism is most plausibly relevant at the graft interface during reperfusion phase and in the context of ischemia–reperfusion injury, when donor endothelial glycoalyx and composition of glycans are rapidly remodeled. However, its quantitative contribution relative to dominant MHC class I–dependent NK-cell regulation remains to be defined directly in transplant models.

#### 4.3.3 Cross-talk between donor glycans and NK siglecs during reperfusion

The reperfusion phase provides a critical window in which endothelial glycan landscapes shift rapidly. Inflammatory activation, neuraminidase activity, oxidative stress, and metabolic disruption collectively reduce sialylation, altering the balance of activating versus inhibitory NK signals. The loss of Siglec ligands not only permits NK-cell activation but also synergizes with the exposure of activating stress ligands, compounding graft vulnerability (100). These interactions suggest that NK cells serve as early sentinels of glycan integrity at the graft-blood interface.

### 4.4 Conceptual relevance for organ engineering

The mechanistic principles governing NK-cell recognition have substantial implications for organ engineering design. As the preceding sections illustrate, NK checkpoints are fundamentally glycan-gated, defined not by a single ligand–receptor interaction but by a composite of sialylation density, multivalency, glycan topology, and Siglec engagement. These same rules apply in bioengineered tissues, where the immune system assesses glycan architecture as an early indicator of graft fitness.

A central concept is sialylation-mediated inhibitory tone. A graft with organized, high-density sialylation can engage Siglec-7 and Siglec-9, promoting an inhibitory milieu that suppresses NK activation. This “sialylation tone” acts as a calibrator of NK and memory T cell responsiveness, influencing early graft survival. Conversely, when sialylation patterns are degraded or disorganized, the inhibitory input from Siglecs declines, lowering NK and memory T cell activation thresholds and predisposing the graft to early cytotoxic responses (Figure 4D).

A second key principle is the balance between masked vs. exposed activating ligands. NK sensitivity to ligand masking highlights the importance of glycan architecture: proper glycan shielding reduces exposure of NKp46- and NKG2D-activating ligands, whereas desialylated or structurally disrupted glycans unmask these danger signals.

Finally, Siglec thresholds for NK hypoactivation underscore that NK activation depends on whether activating signals surpass a threshold shaped by inhibitory Siglec input. Mechanistically, this means that NK cell behavior in transplantation is dictated by relative, not absolute, signaling, a principle that elevates glycan structure as a central determinant of NK-cell restraint or activation.

Rather than proposing specific engineering approaches, the focus here is on the immune logic: NK cell recognition is deeply intertwined with glycan context, making glycan design a conceptual lever for future immune-compatible organ or cell engineering. Incorporating the rules of sialylation density, glycan topology, ligand masking, and Siglec-mediated inhibition into organ bioengineering may therefore provide a path toward more predictable, immune-quiescent grafts.

## 5 Complement-mediated glycan immune checkpoints: factor H as a sialic acid-dependent regulator

Although NK-cell biology represents a major axis of glycan-dependent innate immune regulation, the complement system operates in parallel as a second pathway that interprets the glycan landscape of transplanted tissues. At the center of this pathway is FH, the primary negative regulator of the alternative complement pathway, which distinguishes host-like versus foreign surfaces largely through sialic acid–dependent binding (60). FH recognizes *α*2–3 and *α*2–6 sialylated motifs, as well as polyanionic glycosaminoglycans, enabling it to localize to self-tissues and suppress complement amplification. This includes accelerating the decay of the C3 convertase and promoting cleavage of C3b into its inactive forms.

In the context of transplantation, the graft's sialylation profile becomes a critical determinant of complement susceptibility. Desialylation, oxidative glycan remodeling, glycocalyx shedding and exposure of underlying glycan structures due to IRI sharply reduces FH binding. Without adequate FH recruitment, the alternative pathway proceeds unchecked, generating robust C3 deposition, opsonization, and early endothelial injury (58). Xenogeneic tissues are particularly vulnerable: species-specific differences in sialic acid composition (e.g., Neu5Gc expression or reduced human-compatible *α*2–6 sialylation) impair FH recruitment from the outset, predisposing xenografts to hyperacute complement attack (27, 51). Crucially, complement dysregulation does not occur in isolation. Instead, complement activation shapes, and is shaped by, NK-cell responses, forming a coordinated glycan-sensing network.

In addition, complement activation amplifies NK cell recruitment and activation. For instance, C3a and C5a produced during complement activation act as chemoattractants, drawing NK cells into the graft and increasing local inflammation. Complement deposition on endothelial surfaces further enhances NK engagement by creating a “danger-marked” landscape (Figures 4A,D) (47). Complement attack promotes endothelial stress, cytoskeletal disruption, and surface remodeling (e.g., glycocalyx shedding), conditions that increase the accessibility of NKG2D ligands, NKp46 ligands, and desialylated glycoproteins. These shifts potentiate NK activation in a feed-forward loop (95). These glycan perturbations not only weaken FH binding but can also weaken Siglec-mediated inhibition. Loss of sialylation simultaneously reduces FH recruitment to the graft surface and diminishes the engagement of Siglec-7 and Siglec-9 on NK cells. As a result, the graft experiences a coordinated collapse of inhibitory tone across both complement and NK-cell pathways, lowering innate activation thresholds and amplifying early inflammatory injury (41).

Complement activation can program the graft endothelium during alloimmune injury. In antibody-mediated and mixed rejection, donor-specific antibodies bind graft microvascular endothelium and activate complement. Complement is increasingly recognized as functioning not only as a terminal lytic effector system, but also as a source of non-lytic signals that can reprogram endothelial cells. Complement deposition on endothelial cells can trigger non-lytic signaling that induces a proinflammatory gene program, upregulates leukocyte adhesion and costimulatory cues, and amplifies local T cell recruitment, thereby promoting chronic vascular lesions such as cardiac allograft vasculopathy. This endothelial signaling axis has been defined in key experimental transplant studies, including work demonstrating that alloantibody and complement deposition activate noncanonical NF kappa B signaling in graft endothelium and enhance downstream alloreactive T cell responses. Complement blockade at the level of C5 attenuates antibody and ischemia reperfusion-driven vascular lesions and T cell infiltration in transplant models, reinforcing the idea that complement activation on the endothelial surface can shape adaptive alloimmunity, not only execute injury by its lytic action (18, 43).

Surfaces capable of binding FH tend to maintain a homeostatic glycan profile, rich in *α*2–6 sialylation, thereby preserving Siglec engagement resulting in restraint of NK-cell meditated cytotoxicity. Thus, complement regulation and NK inhibition emerge from a shared glycan logic (114).

These points underscore the point that sialylation acts as a unifying molecular determinant across complement and NK-cell checkpoints. In transplantation, the degree to which graft surfaces can present “FH-compatible” and “Siglec-compatible” sialic acid motifs may dictate the trajectory of early innate injury. NK-cell cytotoxicity and complement-mediated damage are therefore not independent barriers but intersecting glycan-governed processes that collectively define the immune landscape during graft reperfusion.

## 6 IRI-induced glycocalyx collapse as an upstream trigger of alloimmune activation

Another mechanistic checkpoint relevant to transplantation is the structural integrity of the endothelial glycocalyx during ischemia–reperfusion injury. IRI triggers rapid shedding of sialylated glycoproteins, heparan sulfate, and proteoglycans, resulting in the loss of the organized glycan barrier that normally engages Siglec receptors and recruits complement regulator Factor H (25, 82). This injury state can be viewed as a “glycan switch,” in which a self-like, inhibitory endothelial interface is rapidly converted into an exposed, lectin- and complement-reactive surface. This collapse both diminishes inhibitory signals and exposes underlying mannose-rich and desialylated motifs that can activate CLRs, complement, and NK cell receptors. The shift is therefore not merely quantitative but architectural: the destruction of glycan microdomains is predicted to disrupt inhibitory clustering and accelerate immunogenic pattern exposure (Figure 4D).

Restoring or stabilizing glycocalyx architecture may thus represent a critical glycoengineering goal in transplantation. Maintaining structural glycan density during procurement, perfusion, or *ex vivo* organ culture could preserve Siglec engagement, reduce complement amplification, and prevent early innate immune escalation. Because IRI-driven glycocalyx collapse/shedding occurs upstream of antigen release, antigen handling, and lymphoid priming, it provides a mechanistic bridge to the glycan-dependent regulation of direct, indirect, and semi-direct allorecognition described next.

## 7 Relationship between glycan checkpoints and classical allorecognition

Glycan-mediated immune regulation should not be viewed as replacing classical transplant immunology paradigms. Peptide–MHC mismatch, costimulatory signaling, donor-specific antibodies, complement activation, and inflammatory cytokines remain central determinants of graft rejection (18, 43, 75). Rather, glycan architecture modifies how these protein-centric pathways are initiated, amplified, and sustained by shaping early innate activation thresholds, endothelial injury, antigen uptake, APC maturation, complement amplification, and lymph-node priming (1, 25, 63, 80).

Temporally, glycan-dependent recognition is likely most influential during the early phase of transplantation including organ procurement, preservation, reperfusion, and the early innate immune activation, when endothelial glycocalyx integrity, sialylation density, Factor H recruitment, CLR engagement, and NK-cell activation thresholds are rapidly altered (9, 21, 25, 99). However, glycan-mediated responses may differ from protein antigen–driven allorecognition in one important respect: in ABO-incompatible transplantation, grafts can develop accommodation, a state in which the graft continues to function despite the persistence or rebound of anti-A/B antibodies. This phenomenon is well described for carbohydrate blood-group antigens but is not generally recognized for protein alloantigens such as HLA, underscoring a distinctive feature of transplant glycan immunology relevant to hyperacute, acute antibody-mediated, and chronic rejection (64, 74, 79). In contrast, peptide–MHC recognition, co-stimulation, clonal T-cell expansion, donor-specific antibody production, and chronic rejection mechanisms dominate later adaptive phases (12, 75, 107). Thus, the glycan layer may be most consequential early, by setting the inflammatory tone in which direct, indirect, and semi-direct allorecognition subsequently unfold.

Quantitatively, glycan-mediated effects are unlikely to override strong HLA mismatch or potent donor-specific antibody responses on their own (55). Instead, they may shift activation thresholds, alter antigen dose and routing, influence the quality of APC maturation, limit DAMP-driven inflammatory priming after endothelial injury, and modify the balance between inhibitory and activating innate signals (87, 89, 93, 101). Consistent with this threshold-modifying role, endothelial glycocalyx rebuilding in vascular allografts reduced anti-allograft antibody generation, potentially by limiting endothelial injury, DAMP release, and APC priming (93). This positions glycans as modulators of alloimmune intensity rather than independent substitutes for classical protein-mediated pathways.

## 8 Glycan-dependent regulation of alloimmune activation

**Figure 5:**
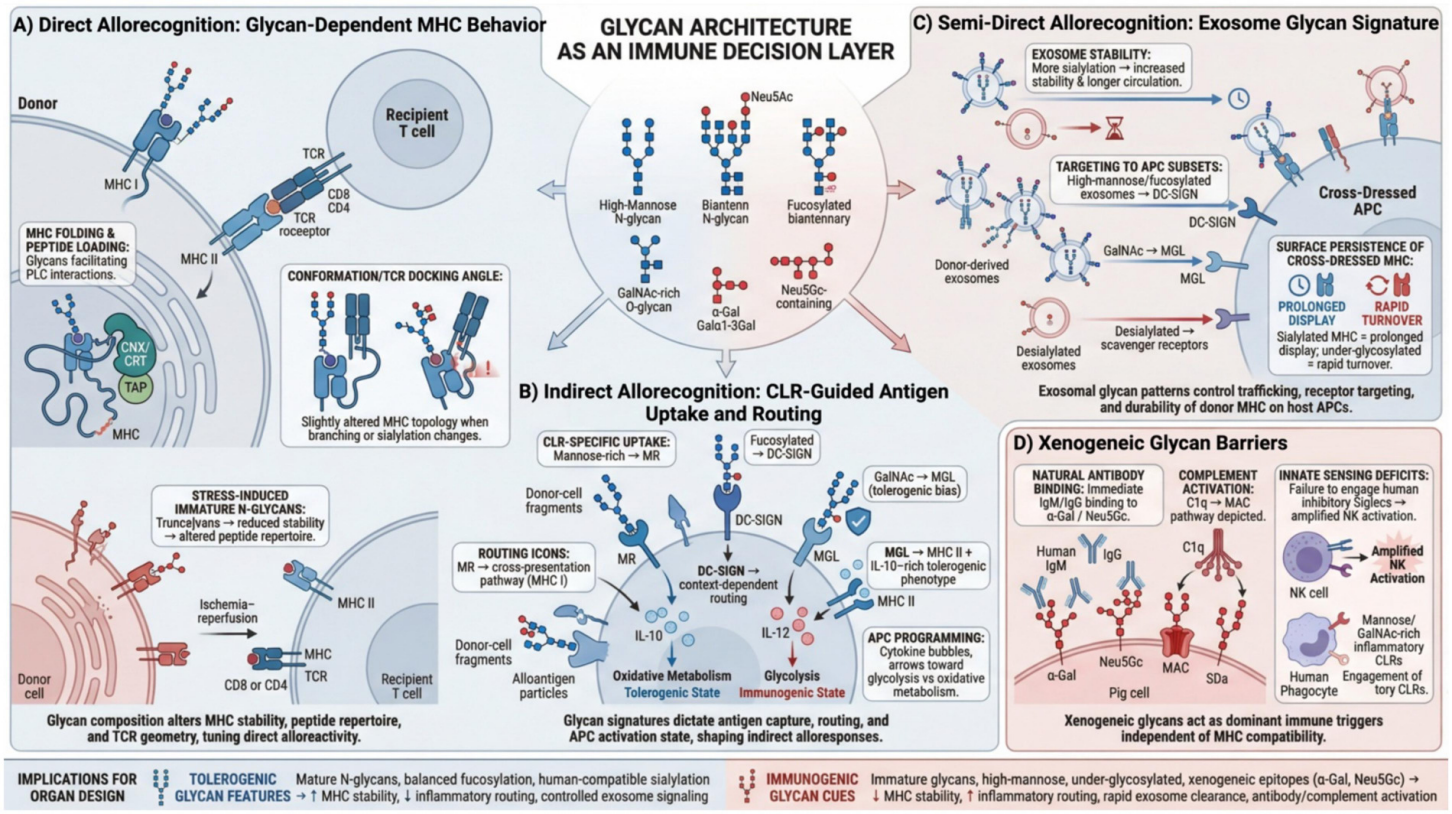
Glycan architecture as a regulatory layer across direct, indirect, and semi-direct allorecognition pathways. **(A)** Direct allorecognition is shaped by glycan-dependent effects on MHC folding, peptide loading, stability, and TCR docking geometry; stress-induced immature N-glycans alter peptide repertoires and enhance direct alloresponses. **(B)** In the indirect pathway, donor glycan motifs guide CLR-mediated antigen uptake and routing, driving tolerogenic (IL-10, oxidative metabolism) or immunogenic (IL-12, glycolysis) APC programming. **(C)** In the semi-direct pathway, exosomal glycan signatures determine vesicle stability, targeting to specific APC subsets, and the persistence of cross-dressed donor MHC on recipient APCs. **(D)** Xenogeneic glycans (*α*-Gal, Neu5Gc, SDa) act as dominant immune triggers through natural antibody binding, complement activation, and innate sensing mismatches. Collectively, glycan composition modulates multiple layers of allorecognition and represents a key parameter for designing immune-compatible organs.

Allorecognition, whether direct, indirect, or semi-direct, forms the core of adaptive immune responses to transplanted organs (Figure 5). Although these pathways are conventionally framed in terms of peptide–MHC mismatches and T-cell receptor (TCR) specificity, the glycan biology of both donor and recipient cells profoundly shapes the efficiency, stability, and immunogenicity of these processes. Importantly, the strength of evidence linking specific glycan mechanisms to allorecognition in transplantation is uneven: some observations derive from transplant/IRI settings, whereas others are mechanistic principles established in non-transplant models (e.g., infection, cancer, or fundamental antigen-presentation systems). In the sections below, we therefore distinguish findings supported in transplant contexts from concepts that are extrapolated and presented as testable hypotheses.

### 8.1 Direct allorecognition modulated by MHC glycosylation

Direct allorecognition occurs when recipient T cells recognize intact donor peptide-MHC molecules on graft cells. Because MHC class I and II molecules are themselves glycoproteins, their glycan composition and branching patterns can influence nearly every step of MHC biology, from folding to trafficking to TCR engagement.

#### 8.1.1 MHC folding, stability, and peptide loading

N-Glycans on MHC class I heavy chains and on class II *α*/*β* chains play a critical role in stabilizing MHC folding intermediates and supporting interactions with chaperones such as calnexin (CNX), calreticulin (CRT), and tapasin (TAP) (Figure 5A). Altered glycan branching or incomplete glycosylation can reduce MHC stability, modify peptide loading efficiency, and shift the distribution of presented peptides. This influences the peptide–loading complex (PLC) and may bias the repertoire of peptides displayed to recipient T cells (23).

#### 8.1.2 Conformation and TCR docking geometry

 Glycan heterogeneity, whether in branching, sialylation, or fucosylation, can alter HLA/MHC surface presentation and microenvironment and can reshape the conformational ensemble of glycosylated TCR–pMHC complexes in silico, with corresponding effects on predicted bond lifetimes and interfacial contacts (84). In the direct pathway, where TCRs often engage allo-MHC with unusually high affinity, such glycan-dependent shifts could plausibly modulate recognition thresholds in a clone- and allele-dependent manner. Notably, evidence that class I glycosylation can influence alloreactive CTL recognition exists but is historically mixed, some classic studies report a requirement for native class I glycosylation for allogeneic CTL recognition, whereas others find carbohydrate moieties dispensable for CTL recognition of class I alloantigens (5, 31). Accordingly, in allo-transplant settings, MHC glycosylation should be viewed as a context-dependent modifier of direct alloreactivity rather than a universal determinant of alloreactive T-cell recognition, and as a testable mechanism for donor–recipient variation beyond allelic mismatch.

#### 8.1.3 Glycan remodeling under stress

IRI or cellular stress can alter MHC glycosylation, leading to reduced glycan maturation and exposure of immature N-glycans. These forms may reduce MHC stability or shift peptide repertoires, increasing the likelihood that T cells perceive donor cells as “altered self,” thereby enhancing direct alloreactivity (32, 109). Here, the evidence base is strongest for stress/IRI altering glycosylation and antigen presentation capacity in general, while the specific contribution to *direct allorecognition in vivo* remains to be defined (25, 86).

### 8.2 Indirect allorecognition influenced by APC glycan biology

In the indirect pathway, recipient APCs process donor antigens and present these donor peptides on self-MHC molecules. Glycan-mediated pathways are most relevant upstream of TCR engagement, where donor glycan motifs and CLR biology can influence graft-antigen capture, intracellular routing, APC maturation, costimulatory state, and cytokine context before peptide–MHC recognition occurs. Glycocalyx preservation may also reduce DAMP-driven APC conditioning after endothelial injury, thereby linking endothelial surface protection to weaker indirect allorecognition and downstream humoral responses.

#### 8.2.1 CLR-mediated antigen uptake

Recipient dendritic cells and macrophages express a variety of CLRs (DC-SIGN, MR, MGL), each with distinct glycan specificities. DC-SIGN preferentially recognizes high-mannose N-glycans and branched fucosylated motifs (e.g., Lewis-type structures), supporting efficient capture of fucosylated/mannosylated cargo at the cell surface (30). MR (CD206) is a highly recycling endocytic receptor that internalizes mannose-rich ligands via clathrin-dependent uptake and can deliver cargo to endosomal compartments compatible with antigen processing and, in some contexts, cross-presentation (101) (Figure 5B). Consistent with these specificities, donor alloantigens displaying mannose-rich or fucosylated glycans are expected to be preferentially internalized through MR and/or DC-SIGN, increasing antigen-capture efficiency and potentially biasing subsequent intracellular routing. Notably, DC-SIGN routing is sensitive to ligand/antigen architecture (e.g., multivalency and scaffold), which can shift endosomal trafficking behavior and downstream presentation outcomes (44).

Conversely, antigens enriched in GalNAc motifs may enter MGL-dependent pathways. MGL (CLEC10A/CD301) recognizes terminal GalNAc epitopes (including Tn/sTn and LacdiNAc motifs) and is frequently associated with APC states described as “tolerogenic” in multiple models, including altered cytokine programs and antigen-handling phenotypes (96). These CLR uptake preferences are well supported mechanistically (glycan specificity → uptake → trafficking), but the extent to which donor glycan motifs dictate indirect allorecognition in transplantation likely depends on injury context (e.g., IRI-driven antigen release and APC activation), antigen abundance, and which APC subsets dominate antigen capture and cross-presentation in the draining lymphoid tissues.

#### 8.2.2 Routing to cross-presentation pathways

CLR engagement can determine whether alloantigens are routed to MHC class I cross-presentation pathways or to class II presentation. MR engagement often favors cross-presentation, potentially amplifying CD8+ T-cell priming. DC-SIGN engagement can either promote tolerogenic or immunogenic routing depending on the glycan context, influencing the strength of indirect alloreactivity (29).

#### 8.2.3 Glycan-driven APC programming and peptide processing

Because CLRs regulate cytokine programs and APC metabolism, donor glycan patterns influence how alloantigens are processed. Glycan motifs that trigger IL-10-rich tolerogenic states may reduce costimulation and encourage regulatory T-cell induction. In contrast, mannose-rich or stress-associated glycans favor IL-12 production and glycolysis, enhancing antigen processing and T-cell priming (80). Collectively, these mechanisms support the conclusion that donor glycan landscapes are positioned to condition indirect allorecognition, even though direct causal demonstrations in allo-transplant models remain limited for specific CLR–glycan pairs.

### 8.3 Semi-direct allorecognition and glycan-dependent exosomal transfer

Semi-direct allorecognition occurs when recipient APCs acquire intact donor MHC–peptide complexes (“cross-dressing”) from donor cells through exosomes or trogocytosis (75). Glycosylation critically influences exosome formation, stability, tissue trafficking, and receptor interactions, and therefore may impact the likelihood of cross-dressed APCs initiating T-cell activation (105).

#### 8.3.1 Exosome stability and trafficking

Donor MHC molecules incorporated into exosomes retain their native glycosylation, which affects vesicle stability, resistance to proteolysis, and circulation time. Sialylated exosomes are more stable and have altered biodistribution compared to desialylated or under-glycosylated vesicles (Figure 5C). In a fully MHC-mismatched rat lung transplant model, donor antigen-bearing extracellular vesicles accumulated in mediastinal lymph nodes within hours of engraftment and colocalized with MHC class two positive cells, supporting early delivery of donor material to lymphoid antigen presenting cells (37).

A key point is that extracellular vesicle surface glycans are functional determinants of immune interaction. Systematic glycosidase trimming of extracellular vesicle glycans changes uptake across recipient cell types, consistent with both charge effects and lectin mediated recognition contributing to vesicle internalization (112). *In vivo*, editing sialylation and other surface glycans can also shift vesicle biodistribution and lymph node access, including increased axillary lymph node accumulation after neuraminidase treatment in mouse models (77, 85).

Together, these findings support a conservative transplant framing: donor extracellular vesicles can reach draining lymph nodes early after transplantation, and extracellular vesicle glycosylation can regulate uptake and biodistribution *in vivo*. Donor vesicle glycan state may therefore modulate lymphoid delivery and antigen-presenting cell engagement during semi-direct allorecognition, but its causal relevance in transplant models remains to be tested directly.

#### 8.3.2 Uptake specificity of donor vesicles is strongly shaped by exosomal glycan signatures

Distinct glycans on donor-derived vesicles target them to different APC receptors: DC-SIGN preferentially internalizes high-mannose or fucosylated exosomes, MGL binds GalNAc-rich vesicles, and macrophage scavenger receptors more readily engage desialylated or stress-associated glycoforms (Figure 5C) (50). As a result, the glycan makeup of donor exosomes may bias which recipient APC subsets acquire intact donor MHC complexes, thereby influencing which T-cell populations are subsequently primed. This glycan bias provides a plausible additional layer of immunological specificity to semi-direct allorecognition that cannot be explained by protein-level properties alone.

#### 8.3.3 Cross-dressing efficiency

Because exosomal transfer preserves the glycan structure surrounding donor MHC molecules, glycan integrity may influence how long these complexes remain on the surface of cross-dressed APCs. Stable, sialylated MHC complexes might sustain T-cell stimulation, whereas under-glycosylated complexes may be rapidly internalized or degraded. The efficiency of semi-direct allorecognition is therefore hypothesized to be tied to the glycan-defined fate of exosomal MHC.

### 8.4 T-cell intrinsic glycan checkpoints

In addition to shaping APC behavior and MHC structure, glycans exert direct control over T-cell activation thresholds. N-glycan branching, particularly via *α*-1,6-mannosylglycoprotein 6-beta-N-acetylglucosaminyltransferase A (MGAT5), acts as an intrinsic immune checkpoint by regulating TCR clustering and retention at the immune synapse (40). Increased *β*-1,6-GlcNAc branching stabilizes galectin–glycoprotein lattices, elevating activation thresholds, whereas reduced branching lowers these thresholds and increases T-cell sensitivity to alloantigens (22). These mechanisms are well supported for T-cell signaling generally; their role as quantitative modifiers of alloantigen sensitivity in transplantation is plausible and testable but is not yet uniformly established across graft settings.

Glycosylation also governs the stability and inhibitory function of PD-1 and CTLA-4. Hypoglycosylated PD-1 undergoes rapid internalization, weakening inhibitory tone, while proper glycosylation stabilizes CTLA-4 on regulatory T cells (24). These intrinsic T-cell glycan checkpoints highlight the need to consider glycan-dependent activation logic within recipient T cells when designing tolerogenic grafts.

### 8.5 B-cell and antibody glycan checkpoints

B cells and antibody responses are also controlled by glycan checkpoints. IgG Fc glycans, whose fucosylation, galactosylation, branching, and sialylation modulate Fc*γ* receptor binding, influence the inflammatory potency of donor-specific antibodies (DSAs). Afucosylated IgG enhances antibody-dependent cellular cytotoxicity (ADCC) via Fc*γ*RIIIa, whereas sialylated IgG assumes anti-inflammatory profiles with reduced complement activation (12, 107).

Several glycan epitopes, including *α*-Gal, Neu5Gc, and SDa, act as dominant B-cell antigens that elicit strong natural antibody responses independent of MHC mismatch (Figure 5D) (98). These antibodies activate complement and promote endothelial injury while shaping B-cell priming and memory. B-cell and antibody-centered glyco-immune checkpoints therefore intersect with complement regulation, NK biology, and APC programming, and must be considered in future glycoengineering strategies aimed at mitigating humoral rejection.

### 8.6 Conceptual implications for organ engineering design

Understanding how glycans regulate direct, indirect, and semi-direct allorecognition must be considered in a mechanistic framework for anticipating the immune challenges that future organ designs must navigate. The aim here is not to outline specific glycoengineering tools, but rather to clarify how glycan architecture shapes the immune interpretation of transplanted tissues.

Across these modalities, glycan logic can either potentiate or attenuate immune activation. A graft that presents human-compatible sialylation, balanced fucosylation, and mature N-glycans is more likely to dampen allorecognition by stabilizing donor MHC conformation, biasing APCs toward tolerogenic programming, and supporting controlled exosome trafficking. Conversely, ischemia-induced glycan remodeling or xenogeneic glycoforms can amplify allorecognition by simultaneously destabilizing MHC complexes, enhancing inflammatory antigen routing, and increasing exosome immunogenicity. Thus, glycan patterns represent a central decision-making layer that determines whether allorecognition progresses toward tolerance or activation, and they constitute a critical conceptual axis for designing immune-compatible organs or bioengineered tissues.

## 9 Xenogeneic glycan barriers in mechanistic immunology

Some of the strongest evidence for glycan-dependent immunogenicity comes from xenotransplantation, where non-human glycan epitopes serve as dominant immune triggers. Key xenogeneic glycans include *α*-Gal (Gal*α*1–3Gal*β*1–4GlcNAc), Neu5Gc (a non-human sialic acid), and the SDa antigen (GalNAc-containing glycan) (11). Their immunologic prominence is underscored by the fact that modern donor pigs are engineered to delete the corresponding synthetic pathways and to introduce human complement and coagulation regulators, yet residual antibody binding and complement activation can still emerge *in vivo*.

### 9.1 Natural antibodies and complement activation

Humans possess high-titer natural IgM and IgG against *α*-Gal and Neu5Gc, due to lifelong environmental and dietary exposure. Binding of these antibodies to xenogeneic graft surfaces triggers potent classical pathway complement activation, leading to rapid opsonization, endothelial damage, and graft failure (Figure 5D) (11, 46). Recent pig-to-human kidney xenotransplant studies using transgenic pigs that lack the glycans mentioned above reported immunoglobulin and complement deposition patterns consistent with antibody-mediated xenograft injury (59, 108). As such, even when donor pigs are engineered to reduce immunogenic carbohydrate epitopes and to humanize selected immune regulators, xenografts may still activate innate and adaptive immunity through glycan-dependent mechanisms, including residual or non-canonical antibody targets and complement-mediated vascular injury. These insights demonstrate that glycan signatures constitute a primary barrier, not a secondary modifier, in cross-species transplantation. Importantly, a clinical series demonstrated that C5 inhibition with eculizumab reduced thrombotic microangiopathy in pig-to-human kidney xenotransplantation, reinforcing complement amplification as a modifiable driver of endothelial injury once antibodies engage non-human surface epitopes (45).

### 9.2 Innate sensing through macrophages and NK cells

Xenogeneic glycan landscapes can also bias innate immune cell behavior independently of peptides and MHC. Loss of human-like sialylation patterns and the presence of non-human sialic acids can reduce engagement of inhibitory human Siglecs, while exposure of mannose-rich or GalNAc-terminated motifs can favor lectin-driven uptake and inflammatory programming in antigen-presenting cells. In recent pig-to-human kidney xenotransplantation, multi-omics and immunologic profiling in brain-dead human recipients has identified persistent innate activation signatures alongside evolving humoral responses, consistent with a layered innate plus antibody-driven response even in the context of gene-edited donors (73, 90).

## 10 Toward a mechanistic framework for glycan-regulated immune compatibility

### 10.1 Reconceptualizing the glycan layer as an immune checkpoint system

Traditionally dismissed as a structural coating, the glycan layer is increasingly recognized as a primary immune checkpoint system, operating upstream of protein-level pathways such as HLA presentation, PD-L1–PD-1 interactions, and CD47–SIRP*α* signaling. Glycans act not merely as molecular ornaments but as pre-recognition cues that innate immune cells interpret even before assessing classical receptor–ligand interactions. In this sense, the glycan landscape represents an early, non-redundant filtering mechanism that influences whether subsequent immune pathways interpret a tissue as self, stressed, foreign, or dangerous.

Because immune cells continually sample sialylation density, glycan linkage patterns, fucosylation states, and the exposure of high-mannose or truncated glycans, the glycocalyx effectively encodes a first-pass immunological verdict. A graft presenting organized, human-compatible glycan features may be interpreted as immunologically benign even in the presence of allelic mismatches, whereas a graft displaying desialylated, immature, or xenogeneic glycans may elicit activation despite MHC compatibility or even protein-level immunomodulation. This reframing position the glycan layer as an independent axis of immune control whose structure and dynamics can be engineered, measured, and optimized alongside protein-based strategies.

### 10.2 An integrative model of glycan-driven pathways

**Figure 6:**
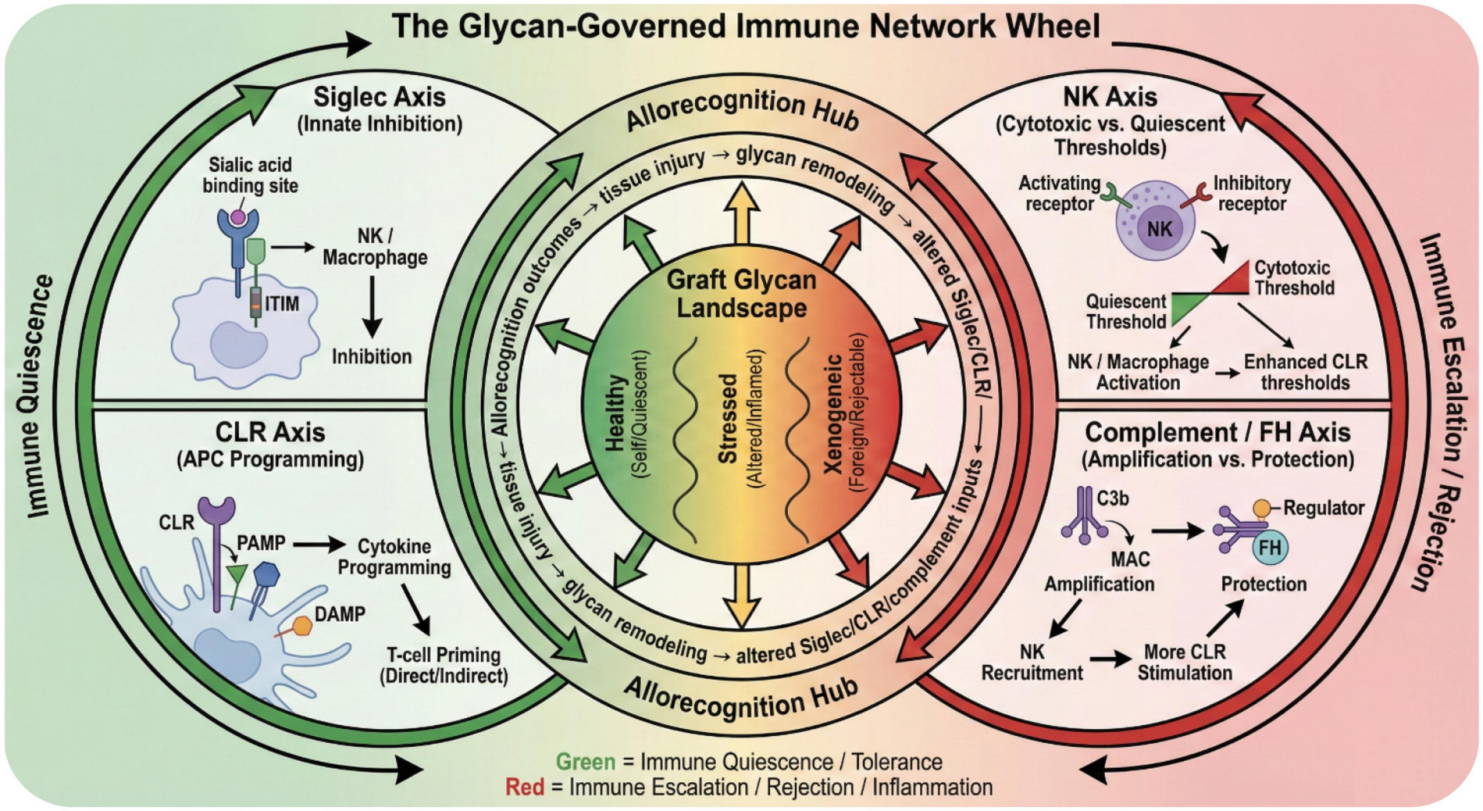
The glycan-governed immune network. Graft glycan states (healthy, stressed, xenogeneic) shape immune outcomes through four interlinked axes: Siglec-mediated inhibition, CLR-driven APC programming, NK cytotoxic thresholds, and complement/Factor H protection. These pathways form reciprocal feedback loops that influence allorecognition and graft fate, with intact glycans supporting immune quiescence (green) and glycan loss driving immune escalation and rejection (red). This network complements classical protein-centric mechanisms of transplant immunity, including peptide–MHC recognition, costimulation, donor-specific antibody binding, cytokine signaling, and checkpoint receptor pathways, by shaping their magnitude, timing, and inflammatory context.

A mechanistic synthesis of the abovementioned sections suggests that glycan-regulated immunity can be conceptualized not as isolated pathways, but as an interconnected network spanning multiple cell types and temporal phases of the host response. Figure 6 summarizes these mechanisms as a coordinated checkpoint network: Siglec-dependent inhibitory tone, CLR-directed antigen handling, NK activation thresholds, Factor H–compatible complement regulation, and downstream allorecognition behave as coupled checkpoints whose outputs reinforce or destabilize one another during reperfusion and early priming. This network view is intended to consolidate the preceding evidence into a single mechanistic logic: glycan architecture influences both the magnitude of early innate escalation and the “framing” of alloantigen for adaptive responses.

### 10.3 Conceptual relevance to bioengineering and transplantation

This Perspective highlights why a mechanistic understanding of glycan-regulated immunity is essential for the rational design of immune-compatible organs for transplantation. If glycans function as a pre-recognition code for innate and adaptive immunity, then any approach to create tolerogenic grafts, whether based on gene editing, stem cell differentiation, perfusion conditioning, decellularization–recellularization, biomaterial scaffolding or cell-surface engineering must account for the glycan landscape it generates.

Importantly, transplant-facing organ engineering studies are beginning to operationalize this principle by treating the endothelial glycocalyx and graft surface carbohydrate landscape as editable interfaces during preservation and perfusion. In our own work, protection of the endothelial glycocalyx with immunosuppressive polymers via cell-surface engineering reduced early immune-cell engagement and prevented vascular allograft rejection, supporting the concept that the luminal glycocalyx can function as a first-line regulatory surface during reperfusion (93). Closely related work from the same broader program has further shown that *ex vivo* engineering of organ blood vessels and vascular allografts can achieve localized immunomodulation at the graft interface (61). Complementing these surface-protection strategies, *ex vivo* enzymatic remodeling of donor-organ blood group antigens in lungs and kidneys demonstrated that graft carbohydrate identity can be deliberately rewritten before transplantation to reduce incompatibility and complement-triggering at reperfusion (65, 106, 118). More broadly, studies of glycocalyx control, complement modulation on engineered cell surfaces, and perfusion-phase graft conditioning support the view that early reperfusion biology is highly sensitive to the molecular architecture of the vascular interface (54, 92). Together, these findings suggest that glycan architecture is a bona fide design variable for vascular protection, complement restraint, and attenuation of innate immune activation.

As a practical design implication, Figure 6 motivates a concise “compatibility checklist” that complements protein-level engineering goals without repeating earlier sections. Engineered grafts should preserve recipient-compatible inhibitory sialylation at the vascular interface, limit injury-associated lectin cues that bias antigen routing toward inflammatory outcomes, maintain glycocalyx integrity to prevent NK-permissive exposure at the synapse, and retain Factor H–compatible surfaces during early reperfusion to restrain complement amplification. Failure at any one node can amplify the others, shifting the system toward inflammation even when protein-level strategies appear optimal.

In this framework, glycan architecture becomes a central parameter of immunity, informing how engineered organs are perceived from the first moments of host–graft contact. By treating glycans as instructive signals rather than passive structures, organ engineering can move toward graft designs that integrate with immune sensing logic rather than merely resisting downstream effector pathways.

### 10.4 Glycan checkpoints in allogenic transplantation and universal donor therapies

Allogeneic cell transplantation is an emerging cell-scale analog of solid organ transplantation: donor-derived immune or progenitor cells must persist in an immunocompetent host while avoiding elimination by host NK cells, macrophages, complement, and alloreactive T cells. As in organ transplantation, the earliest compatibility decisions are often made at the cell surface, where glycocalyx architecture shapes inhibitory versus activating inputs, opsonization and complement amplification, and the likelihood that donor material is transferred to antigen-presenting cells.

Recent studies now provide direct evidence that glycocalyx remodeling can function as an immune-compatibility lever in allogeneic cell therapy. In an allogeneic CAR T context, deletion of signal peptide peptidase-like 3 (SPPL3), a Golgi-resident intramembrane protease that regulates cell-surface glycosylation, conferred glycan-based immune evasion, reduced immune-mediated elimination, and preserved therapeutic activity, supporting the concept that engineered glycan architecture can improve persistence in allogeneic settings (113).

Transplant-facing organ engineering work from our group and collaborators provides a mechanistic parallel by demonstrating that targeted modification of glycan-rich interfaces can reset early immune thresholds at the graft surface. Protecting the endothelial glycocalyx through enzymatic ligation of immunosuppressive polymers attenuated vascular allograft rejection, highlighting surface architecture as a modifiable determinant of innate activation and downstream injury (93). In a complementary organ-glycan editing direction, *ex vivo* enzymatic removal of blood-group antigens has enabled conversion of donor kidneys toward universal compatibility, illustrating that clinically relevant graft surfaces can be glycoengineered during perfusion and preservation (65, 106, 119).

Finally, glycans also shape intercellular communication routes that are relevant to semi-direct allorecognition and to how therapeutic cells engage antigen-presenting cells. A comprehensive review of extracellular vesicle glycosylation summarizes evidence that glycan structures contribute to vesicle formation, cargo loading, stability, and receptor interactions, and that glycan-dependent recognition can bias immune uptake (105). Experimental data further show that enzymatic remodeling of extracellular vesicle glycans alters uptake across recipient cell types, consistent with both lectin-mediated recognition and charge effects contributing to immune interactions (112).

Together, these findings motivate a transplant-parallel design hypothesis for a universal donor: beyond HLA and checkpoint edits, durable allogeneic cell products may also benefit from a glycocalyx architecture that supports inhibitory self-recognition, resists complement amplification, and avoids inflammatory lectin engagement, thereby reducing early innate clearance while preserving therapeutic function.

## 11 Future directions and conclusion

Despite rapid progress in glyco-immunology, several mechanistic gaps limit the ability to predict or modulate how transplanted tissues are interpreted by the immune system. One key challenge is the incomplete understanding of glycan spatial topology, glycan multivalency, the nanoscale organization, density, and microdomain clustering on cell surface that determine whether Siglecs, CLRs, NK cell receptors, and complement regulators can engage their ligands effectively. Current analyses rely largely on bulk glycomics, which cannot resolve the topological patterns that immune cells sense. Equally unresolved are the quantitative thresholds that govern glycan-based gating *in vivo*. It remains unclear how much sialylation is required to sustain Siglec-mediated inhibition, which patterns of desialylation are sufficient to unmask NK-activating ligands, and how glycocalyx collapse/shedding during ischemia–reperfusion reshapes these thresholds in time and space at the vascular interface. Similarly, donor–recipient variability in CLR signaling thresholds and antigen-routing pathways is poorly understood. Addressing these gaps will require *in situ* glycomics and spatial glycoprofiling of engineered organs and donor tissues, paired with perturbation experiments that deliberately tune glycan density, linkage, and architecture and read out innate escalation and priming outcomes.

These scientific challenges present opportunities for deeper cross-disciplinary integration. Organoid biology and advanced perfusion systems provide platforms for mapping how glycan remodeling unfolds under ischemia–reperfusion stress or mechanical conditioning. Transplant immunology models can incorporate glycan variation to determine how innate and adaptive pathways respond to controlled perturbations of sialylation, fucosylation, or mannose exposure. *Ex vivo* organ perfusion offers a particularly tractable translational setting to test causal glycan edits at the graft surface immediately before implantation, enabling direct linkage between engineered glycan states and early reperfusion biology. Meanwhile, computational modeling offers an emerging avenue for predicting immune trajectories from glycan patterns. Mechanistic models that incorporate Siglec engagement kinetics, CLR routing logic, NK-synapse geometry, and glycan-dependent complement regulation could enable in silico predictions of tolerance versus activation. Integrating these disciplines may ultimately reveal a unified framework in which glycan signatures can be interpreted as immune “states” that predict graft behavior.

A mechanistic understanding of glycan–immune interactions may enable rational organ design, allowing engineered tissues to present glycan cues that promote quiescence, suppress inflammatory routing, and reduce reliance on systemic immunosuppression. Glycan-based immunoregulation has the potential to create more predictable and durable tolerance by shaping immune interpretation at the earliest stages of host–graft interaction. Notably, the same logic is now becoming relevant beyond solid organs: emerging allogeneic cell therapies face parallel compatibility constraints, and early work suggests that engineered surface glycan architectures can influence immune persistence, highlighting glycans as a shared control layer across transplant modalities.

Siglec-mediated inhibition, CLR-driven antigen programming, NK-cell glycan sensing, and glycan-governed allorecognition collectively shape how transplanted tissues are classified and responded to by the host immune system. Understanding these mechanisms is essential for moving beyond empirical immunosuppression toward precision-guided immune compatibility. This Perspective establishes a conceptual basis for integrating glycan immunology with emerging organ engineering approaches while remaining distinct from prior glycocalyx-focused reviews. By reframing the glycan layer as an instructive and actively interpreted biological code, we lay the groundwork for future innovations in transplantation and in the design of universal donor platforms spanning engineered organs and allogeneic cell products.

## References

[1] Abassi Z Armaly Z Heyman SN. Glycocalyx degradation in ischemia-reperfusion injury. Am J Pathol. (2020) 190:752–67. 10.1016/j.ajpath.2019.08.019 32035883

[2] Almeida P Alves I Fernandes Â Lima C Freitas R Braga I. Mannose glycans as key players in trained immunity: a novel anti-tumoral catalyst. Biochimica et Biophysica Acta (BBA) - General Subjects. (2025) 1869:130779. 10.1016/j.bbagen.2025.130779 39988110

[3] Alves I Fernandes Â Santos-Pereira B Azevedo CM Pinho SS. Glycans as a key factor in self and nonself discrimination: impact on the breach of immune tolerance. FEBS Lett. (2022) 596:1485–502. 10.1002/1873-3468.14347 35383918

[4] Arkatkar T Davé V Talavera IC Graham JB Swarts JL Hughes SM. Memory T cells possess an innate-like function in local protection from mucosal infection. J Clin Invest. (2023) 133:e162800. 10.1172/JCI162800 36951943 PMC10178838

[5] Bagriaçik EU Kirkpatrick A Miller KS. Glycosylation of native MHC class Ia molecules is required for recognition by allogeneic cytotoxic T lymphocytes. Glycobiology. (1996) 6:413–21. 10.1093/glycob/6.4.413 8842705

[6] Banerjee S Mwangi JG Stanley TK Mitra R Ebong EE. Regeneration and assessment of the endothelial glycocalyx to address cardiovascular disease. Ind Eng Chem Res. (2021) 60:17328–47. 10.1021/acs.iecr.1c03074

[7] Barkal AA Brewer RE Markovic M Kowarsky M Barkal SA Zaro BW. CD24 Signalling through macrophage siglec-10 is a target for cancer immunotherapy. Nature. (2019) 572:392–6. 10.1038/s41586-019-1456-0 31367043 PMC6697206

[8] Batal I Mohan S De Serres SA Vasilescu E-R Tsapepas D Crew RJ. Analysis of dendritic cells and ischemia-reperfusion changes in postimplantation renal allograft biopsies may serve as predictors of subsequent rejection episodes. Kidney Int. (2018) 93:1227–39. 10.1016/j.kint.2017.12.015 29544662

[9] Blaum BS Hannan JP Herbert AP Kavanagh D Uhrín D Stehle T. Structural basis for sialic acid–mediated self-recognition by complement factor H. Nat Chem Biol. (2015) 11:77–82. 10.1038/nchembio.1696 25402769

[10] Borges TJ Lima K Gassen RB Liu K Ganchiku Y Ribas GT. The inhibitory receptor Siglec-E controls antigen-presenting cell activation and T cell–mediated transplant rejection. Sci Transl Med. (2025) 17:eads2694. 10.1126/scitranslmed.ads2694 40333992 PMC12135932

[11] Byrne GW McGregor CGA. 2025: status of cardiac xenotransplantation including preclinical models. Front Transplant. (2025) 4:1568910. 10.3389/frtra.2025.1568910 40302932 PMC12037586

[12] Cambay F Henry O Durocher Y De Crescenzo G. Impact of N-glycosylation on fc*γ* receptor/IgG interactions: unravelling differences with an enhanced surface plasmon resonance biosensor assay based on coiled-coil interactions. MAbs. (2019) 11:435–52. 10.1080/19420862.2019.1581017 30822189 PMC6512902

[13] Cao H Crocker PR. Evolution of CD33-related siglecs: regulating host immune functions and escaping pathogen exploitation? Immunology. (2011) 132:18–26. 10.1111/j.1365-2567.2010.03368.x 21070233 PMC3015071

[14] Castenmiller C Keumatio-Doungtsop B-C van Ree R de Jong EC van Kooyk Y. Tolerogenic immunotherapy: targeting DC surface receptors to induce antigen-specific tolerance. Front Immunol. (2021) 12:643240. 10.3389/fimmu.2021.643240 33679806 PMC7933040

[15] Chao Z Mei Q Yang C Luo J Liu P Peng H. Immunological synapse: structures, molecular mechanisms and therapeutic implications in disease. Sig Transduct Target Ther. (2025) 10:254. 10.1038/s41392-025-02332-6 PMC12336355 40784895

[16] Chen R Zou J Chen J Zhong X Kang R Tang D. Pattern recognition receptors: function, regulation and therapeutic potential. Sig Transduct Target Ther. (2025) 10:216. 10.1038/s41392-025-02264-1 PMC12246121 40640149

[17] Chin-Hun Kuo J Gandhi JG Zia RN Paszek MJ. Physical biology of the cancer cell glycocalyx. Nat Phys. (2018) 14:658–69. 10.1038/s41567-018-0186-9 33859716 PMC8046174

[18] Chun N Horwitz J Heeger PS. Role of complement activation in allograft inflammation. Curr Transplant Rep. (2019) 6:52–9. 10.1007/s40472-019-0224-2 31673484 PMC6822566

[19] Cummings RD. The mannose receptor ligands and the macrophage glycome. Curr Opin Struct Biol. (2022) 75:102394. 10.1016/j.sbi.2022.102394 35617912 PMC10243190

[20] Dai H Friday AJ Abou-Daya KI Williams AL Mortin-Toth S Nicotra ML. Donor SIRP*α* polymorphism modulates the innate immune response to allogeneic grafts. Sci Immunol. (2017) 2:eaam6202. 10.1126/sciimmunol.aam6202 28783664 PMC5653256

[21] Daly J Carlsten M O’Dwyer M. Sugar free: novel immunotherapeutic approaches targeting siglecs and sialic acids to enhance natural killer cell cytotoxicity against cancer. Front. Immunol. (2019) 10:1047. 10.3389/fimmu.2019.01047 31143186 PMC6521797

[22] de-Souza-Ferreira M Ferreira ÉE de-Freitas-Junior JCM. Aberrant N-glycosylation in cancer: mGAT5 and *β*1,6-GlcNAc branched N-glycans as critical regulators of tumor development and progression. Cell Oncol (Dordr). (2023) 46:481–501. 10.1007/s13402-023-00770-4 36689079 PMC12974675

[23] Domnick A Winter C Sušac L Hennecke L Hensen M Zitzmann N. Molecular basis of MHC I quality control in the peptide loading complex. Nat Commun. (2022) 13:4701. 10.1038/s41467-022-32384-z 35948544 PMC9365787

[24] Duan Z Shi R Gao B Cai J. N-linked glycosylation of PD-L1/PD-1: an emerging target for cancer diagnosis and treatment. J Transl Med. (2024) 22:705. 10.1186/s12967-024-05502-2 39080767 PMC11290144

[25] Duni A Liakopoulos V Koutlas V Pappas C Mitsis M Dounousi E. The endothelial glycocalyx as a target of ischemia and reperfusion injury in kidney transplantation—where have we gone so far? Int J Mol Sci. (2021) 22:2157. 10.3390/ijms22042157 33671524 PMC7926299

[26] Feinberg H Jégouzo SAF Lasanajak Y Smith DF Drickamer K Weis WI. Structural analysis of carbohydrate binding by the macrophage mannose receptor CD206. J Biol Chem. (2021) 296:100368. 10.1016/j.jbc.2021.100368 33545173 PMC7949135

[27] French BM Sendil S Pierson RN III Azimzadeh AM. The role of sialic acids in the immune recognition of xenografts. Xenotransplantation. (2017) 24:e12345. 10.1111/xen.12345 PMC10167934 29057592

[28] Garcia de Mattos Barbosa M Cascalho M Platt JL. Accommodation in ABO-incompatible organ transplants. Xenotransplantation. (2018) 25:e12418. 10.1111/xen.12418 29913044 PMC6047762

[29] García-Vallejo JJ Unger WWJ Kalay H van Kooyk Y. Glycan-based DC-SIGN targeting to enhance antigen cross-presentation in anticancer vaccines. OncoImmunology. (2013) 2:e23040. 10.4161/onci.23040 23525136 PMC3601176

[30] Geurtsen J Driessen NN Appelmelk BJ. “Mannose–fucose recognition by DC-SIGN”. In: Holst O Brennan PJ von Itzstein M, editors. Microbial Glycobiology. Amsterdam: Academic Press, an imprint of Elsevier (2010). p. 673–95. 10.1016/B978-0-12-374546-0.00034-1

[31] Goldstein SA Mescher MF. Carbohydrate moieties of major histocompatibility complex class I alloantigens are not required for their recognition by T lymphocytes. J Exp Med. (1985) 162:1381–6. 10.1084/jem.162.4.1381 3876403 PMC2187872

[32] Granados DP Tanguay P-L Hardy M-P Caron É de Verteuil D Meloche S. ER Stress affects processing of MHC class I-associated peptides. BMC Immunol. (2009) 10:10. 10.1186/1471-2172-10-10 19220912 PMC2657905

[33] Groh V Rhinehart R Randolph-Habecker J Topp MS Riddell SR Spies T. Costimulation of CD8alphabeta T cells by NKG2D via engagement by MIC induced on virus-infected cells. Nat Immunol. (2001) 2:255–60. 10.1038/85321 11224526

[34] Gubbels JA Felder M Horibata S Belisle JA Kapur A Holden H. MUC16 provides immune protection by inhibiting synapse formation between NK and ovarian tumor cells. Mol Cancer. (2010) 9:11. 10.1186/1476-4598-9-11 20089172 PMC2818693

[35] Gultom M Rieben R. Complement, coagulation, and fibrinolysis: the role of the endothelium and its glycocalyx layer in xenotransplantation. Transpl Int. (2024) 37:13473. 10.3389/ti.2024.13473 39474588 PMC11518725

[36] Haas Q Boligan KF Jandus C Schneider C Simillion C Stanczak MA. Siglec-9 regulates an effector memory CD8+ T-cell subset that congregates in the melanoma tumor microenvironment. Cancer Immunol Res. (2019) 7:707–18. 10.1158/2326-6066.CIR-18-0505 30988027

[37] Habertheuer A Chatterjee S Sada Japp A Ram C Korutla L Ochiya T. Donor extracellular vesicle trafficking via the pleural space represents a novel pathway for allorecognition after lung transplantation. Am J Transplant. (2022) 22:1909–18. 10.1111/ajt.17023 35285127

[38] Han X Wang M Duan S Franco PJ Kenty JH-R Hedrick P. Generation of hypoimmunogenic human pluripotent stem cells. Proc Natl Acad Sci USA. (2019) 116:10441–6. 10.1073/pnas.1902566116 31040209 PMC6535035

[39] He M Zhou X Wang X. Glycosylation: mechanisms, biological functions and clinical implications. Sig Transduct Target Ther. (2024) 9:194. 10.1038/s41392-024-01886-1 PMC11298558 39098853

[40] Hollander EE Flock RE McDevitt JC Vostrejs WP Campbell SL Orlen M. N-glycosylation by Mgat5 imposes a targetable constraint on immune-mediated tumor clearance. JCI Insight. (2024) 9:e178804. 10.1172/jci.insight.178804 38912584 PMC11383181

[41] Hudak JE Canham SM Bertozzi CR. Glycocalyx engineering reveals a siglec-based mechanism for NK cell immunoevasion. Nat Chem Biol. (2014) 10:69–75. 10.1038/nchembio.1388 24292068 PMC3893890

[42] Ibarlucea-Benitez I Weitzenfeld P Smith P Ravetch JV. Siglecs-7/9 function as inhibitory immune checkpoints *in vivo* and can be targeted to enhance therapeutic antitumor immunity. Proc Natl Acad Sci U S A. (2021) 118:e2107424118. 10.1073/pnas.2107424118 34155121 PMC8256000

[43] Jane-wit D Manes TD Yi T Qin L Clark P Kirkiles-Smith NC. Alloantibody and complement promote T cell-mediated cardiac allograft vasculopathy through non-canonical NF-*κ*B signaling in endothelial cells. Circulation. (2013) 128:2504-16. 10.1161/CIRCULATIONAHA.113.002972 24045046 PMC3885874

[44] Jarvis CM Zwick DB Grim JC Alam MM Prost LR Gardiner JC. Antigen structure affects cellular routing through DC-SIGN. Proc Natl Acad Sci USA. (2019) 116:14862–7. 10.1073/pnas.1820165116 31270240 PMC6660738

[45] Jones-Carr ME Fatima H Kumar V Anderson DJ Houp J Perry JC. C5 inhibition with eculizumab prevents thrombotic microangiopathy in a case series of pig-to-human kidney xenotransplantation. J Clin Invest. (2024) 134:e175996 10.1172/JCI175996 38269581 PMC10904036

[46] Kakuta Y Miyagawa S Matsumura S Higa-Maegawa Y Fukae S Tanaka R. Complement and complement regulatory protein in allogeneic and xenogeneic kidney transplantation. Transplant Rev. (2025) 39:100885. 10.1016/j.trre.2024.100885 39536474

[47] Kanbay M Ozbek L Guldan M Akcin Z Susal CC Yilmaz AM. Complement activation in kidney transplantation. Nephrol Dial Transplant. (2025) 41:816. 10.1093/ndt/gfaf206 PMC13161900 41042248

[48] Kim T-S Shin E-C. The activation of bystander CD8+ T cells and their roles in viral infection. Exp Mol Med. (2019) 51:1–9. 10.1038/s12276-019-0316-1 PMC6906361 31827070

[49] Kononova S Litvinova E Vakhitov T Skalinskaya M Sitkin S. Acceptive immunity: the role of fucosylated glycans in human host–microbiome interactions. Int J Mol Sci. (2021) 22:3854. 10.3390/ijms22083854 33917768 PMC8068183

[50] Kuipers ME Nolte-‘t Hoen ENM van der Ham AJ Ozir-Fazalalikhan A Nguyen DL de Korne CM. DC-SIGN mediated internalisation of glycosylated extracellular vesicles from schistosoma mansoni increases activation of monocyte-derived dendritic cells. J Extracell Vesicles. (2020) 9:1753420. 10.1080/20013078.2020.1753420 32489529 PMC7241508

[51] Lee H Park EM Ko N Choi K Oh KB Kang HJ. Effect of factor H on complement alternative pathway activation in human Serum remains on porcine cells lacking N-glycolylneuraminic acid. Front Immunol. (2022) 13:859261. 10.3389/fimmu.2022.859261 35444661 PMC9014258

[52] Lee J Sheen JH Lim O Lee Y Ryu J Shin D. Abrogation of HLA surface expression using CRISPR/Cas9 genome editing: a step toward universal T cell therapy. Sci Rep. (2020) 10:17753. 10.1038/s41598-020-74772-9 33082438 PMC7576162

[53] Lee RT Hsu T-L Huang SK Hsieh S-L Wong C-H Lee YC. Survey of immune-related, mannose/fucose-binding C-type lectin receptors reveals widely divergent sugar-binding specificities. Glycobiology. (2011) 21:512–20. 10.1093/glycob/cwq193 21112966 PMC3055596

[54] Leung VL Kizhakkedathu JN. The mechanism and modulation of complement activation on polymer grafted cells. Acta Biomater. (2016) 31:252–63. 10.1016/j.actbio.2015.11.022 26593783

[55] Li Q Lan P. Activation of immune signals during organ transplantation. Sig Transduct Target Ther. (2023) 8:110. 10.1038/s41392-023-01377-9 PMC10008588 36906586

[56] Lin S-Y Schmidt EN Takahashi-Yamashiro K Macauley MS. Roles for Siglec-glycan interactions in regulating immune cells. Semin Immunol. (2025) 77:101925. 10.1016/j.smim.2024.101925 39706106

[57] Linke A Cicek H Müller A Meyer-Schwesinger C Melderis S Wiech T. Antigen cross-presentation by murine proximal tubular epithelial cells induces cytotoxic and inflammatory CD8+ T cells. Cells. (2022) 11:1510. 10.3390/cells11091510 35563816 PMC9104549

[58] Loeven MA Rops AL Lehtinen MJ van Kuppevelt TH Daha MR Smith RJ. Mutations in complement factor H impair alternative pathway regulation on mouse glomerular endothelial cells *in vitro**. J Biol Chem. (2016) 291:4974–81. 10.1074/jbc.M115.702506 26728463 PMC4777835

[59] Loupy A Goutaudier V Giarraputo A Mezine F Morgand E Robin B. Immune response after pig-to-human kidney xenotransplantation: a multimodal phenotyping study. Lancet. (2023) 402:1158–69. 10.1016/S0140-6736(23)01349-1 37598688

[60] Lucientes-Continente L Márquez-Tirado B Goicoechea de Jorge E. The factor H protein family: the switchers of the complement alternative pathway. Immunol Rev. (2023) 313:25–45. 10.1111/imr.13166 36382387 PMC10099856

[61] Luo D Siren EM Enns W Sim L Tam F Rahim J. 425.5: achieving localized immunosuppression through *ex vivo* engineering of organ blood vessels. Transplantation. (2022) 106:S486. 10.1097/01.tp.0000888056.30637.25

[62] Luo HD Rana MM Nouri PMM Rahim JF Enns W Wang J-J. Enzymatic Cell-surface Engineering with Glycocalyx Mimicking Anti-Oxidant and Anti-Inflammatory Sulfated Polymers for Protection Against Inflammatory Vascular Injury. Bristol: IOP Publishing Ltd (2026). 10.1088/1748-605X/ae6d62 42127971

[63] Lv D Jiang H Yang X Li Y Niu W Zhang D. Advances in understanding of dendritic cell in the pathogenesis of acute kidney injury. Front Immunol. (2024) 15:1294807. 10.3389/fimmu.2024.1294807 38433836 PMC10904453

[64] Lynch RJ Platt JL. Accommodation in organ transplantation. Curr Opin Organ Transplant. (2008) 13:165. 10.1097/MOT.0b013e3282f6391e 18685298 PMC2726737

[65] MacMillan S Hosgood SA Walker-Panse L Rahfeld P Macdonald SS Kizhakkedathu JN. Enzymatic conversion of human blood group A kidneys to universal blood group O. Nat Commun. (2024) 15:2795. 10.1038/s41467-024-47131-9 38555382 PMC10981661

[66] Malamud M Brown GD. The dectin-1 and dectin-2 clusters: c-type lectin receptors with fundamental roles in immunity. EMBO Rep. (2024) 25:5239–64. 10.1038/s44319-024-00296-2 39482490 PMC11624271

[67] Mantuano NR Läubli H. Sialic acid and siglec receptors in tumor immunity and immunotherapy. Semin Immunol. (2024) 74–75:101893. 10.1016/j.smim.2024.101893 39427573

[68] Mariuzza RA Singh P Karade SS Shahid S Sharma VK. Recognition of self and viral ligands by NK cell receptors. Immunol Rev. (2025) 329:e13435. 10.1111/imr.13435 39748148 PMC11695704

[69] Mathis S Putzer G Schneeberger S Martini J. The endothelial glycocalyx and organ preservation—from physiology to possible clinical implications for solid organ transplantation. Int J Mol Sci. (2021) 22:4019. 10.3390/ijms22084019 33924713 PMC8070558

[70] Meissner TB Schulze HS Dale SM. Immune editing: overcoming immune barriers in stem cell transplantation. Curr Stem Cell Rep. (2022) 8:206–18. 10.1007/s40778-022-00221-0 36406259 PMC9643905

[71] Meyer SJ Linder AT Brandl C Nitschke L. B cell siglecs–news on signaling and its interplay with ligand binding. Front Immunol. (2018) 9:2820. 10.3389/fimmu.2018.02820 30559744 PMC6286995

[72] Min Y Cuevas-Rios G Langmann T Neumann H. Sialylation as a checkpoint for inflammatory and complement-related retinal diseases. Front Cell Neurosci. (2025) 19:1623755. 10.3389/fncel.2025.1623755 40656681 PMC12245909

[73] Montgomery RA Stern JM Fathi F Suek N Kim JI Khalil K. Physiology and immunology of a pig-to-human decedent kidney xenotransplant. Nature. (2026) 650:218–29. 10.1038/s41586-025-09847-6 41233546

[74] Morath C Zeier M Döhler B Opelz G Süsal C. ABO-incompatible kidney transplantation. Front Immunol. (2017) 8:234. 10.3389/fimmu.2017.00234 28321223 PMC5338156

[75] Morelli AE Bracamonte-Baran W Burlingham WJ. Donor-derived exosomes: the trick behind the semidirect pathway of allorecognition. Curr Opin Organ Transplant. (2017) 22:46. 10.1097/MOT.0000000000000372 27898464 PMC5407007

[76] Nawafleh H Zeinelabdin N Greene MK Krishnan A Ho L Genead M. Effect of hypoxia on siglec-7 and siglec-9 receptors and sialoglycan ligands and impact of their targeting on NK cell cytotoxicity. Pharmaceuticals. (2024) 17:1443. 10.3390/ph17111443 39598355 PMC11597189

[77] Nishida-Aoki N Tominaga N Kosaka N Ochiya T. Altered biodistribution of deglycosylated extracellular vesicles through enhanced cellular uptake. J Extracell Vesicles. (2020) 9:1713527. 10.1080/20013078.2020.1713527 32082512 PMC7006786

[78] Oberbarnscheidt MH Zeng Q Li Q Dai H Williams AL Shlomchik WD. Non-self recognition by monocytes initiates allograft rejection. J Clin Invest. (2014) 124:3579–89. 10.1172/JCI74370 24983319 PMC4109551

[79] Park WD Grande JP Ninova D Nath KA Platt JL Gloor JM. Accommodation in ABO-incompatible kidney allografts, a novel mechanism of self-protection against antibody-mediated injury. Am J Transplant. (2003) 3:952–60. 10.1034/j.1600-6143.2003.00179.x 12859529

[80] Pinho SS Alves I Gaifem J Rabinovich GA. Immune regulatory networks coordinated by glycans and glycan-binding proteins in autoimmunity and infection. Cell Mol Immunol. (2023) 20:1101–13. 10.1038/s41423-023-01074-1 37582971 PMC10541879

[81] Raker VK Domogalla MP Steinbrink K. Tolerogenic dendritic cells for regulatory T cell induction in man. Front Immunol. (2015) 6:569. 10.3389/fimmu.2015.00569 26617604 PMC4638142

[82] Rana MM Mohammadi Nouri PM Hosseini SH Roper B Withers SG Kizhakkedathu JN. Reprogramming the glycocalyx: advances in glycoengineering for immunomodulation and regenerative medicine. Biomaterials. (2026) 326:123717. 10.1016/j.biomaterials.2025.123717 40972251

[83] Reis e Sousa C Yamasaki S Brown GD. Myeloid C-type lectin receptors in innate immune recognition. Immunity. (2024) 57:700–17. 10.1016/j.immuni.2024.03.005 38599166

[84] Rollins Z Harris B George S Faller R. A molecular dynamics investigation of N-glycosylation effects on T-cell receptor kinetics. J Biomol Struct Dyn. (2023) 41:5614–23. 10.1080/07391102.2022.2091660 35763488

[85] Royo F Cossío U de Angulo AR Llop J Falcon-Perez JM. Modification of the glycosylation of extracellular vesicles alters their biodistribution in mice. Nanoscale. (2019) 11:1531–7. 10.1039/C8NR03900C 30623961

[86] Ryan SO Cobb BA. Host glycans and antigen presentation. Microbes Infect. (2012) 14:894–903. 10.1016/j.micinf.2012.04.010 22580092 PMC3432721

[87] Saini P Adeniji OS Abdel-Mohsen M. Inhibitory siglec-sialic acid interactions in balancing immunological activation and tolerance during viral infections. eBioMedicine. (2022) 86:104354. 10.1016/j.ebiom.2022.104354 36371982 PMC9663867

[88] Schenk AD Nozaki T Rabant M Valujskikh A Fairchild RL. Donor-reactive CD8 memory T cells infiltrate cardiac allografts within 24-h posttransplant in naive recipients. Am J Transplant. (2008) 8:1652–61. 10.1111/j.1600-6143.2008.02302.x 18557725 PMC2625311

[89] Schetters STT Kruijssen LJW Crommentuijn MHW Kalay H Ochando J den Haan JMM. Mouse DC-SIGN/CD209a as target for antigen delivery and adaptive immunity. Front Immunol. (2018) 9:990. 10.3389/fimmu.2018.00990 29867967 PMC5949514

[90] Schmauch E Piening BD Dowdell AK Mohebnasab M Williams SH Stukalov A. Multi-omics analysis of a pig-to-human decedent kidney xenotransplant. Nature. (2026) 650:205–17. 10.1038/s41586-025-09846-7 41233547 PMC12805800

[91] Scur M Parsons BD Dey S Makrigiannis AP. The diverse roles of C-type lectin-like receptors in immunity. Front Immunol. (2023) 14:1126043. 10.3389/fimmu.2023.1126043 36923398 PMC10008955

[92] Siren EMJ Chapanian R Constantinescu I Brooks DE Kizhakkedathu JN. Oncotically driven control over glycocalyx dimension for cell surface engineering and protein binding in the longitudinal direction. Sci Rep. (2018) 8:7581. 10.1038/s41598-018-25870-2 29765073 PMC5954099

[93] Siren EMJ Luo HD Tam F Montgomery A Enns W Moon H. Prevention of vascular-allograft rejection by protecting the endothelial glycocalyx with immunosuppressive polymers. Nat Biomed Eng. (2021) 5:1202–16. 10.1038/s41551-021-00777-y 34373602

[94] Sobczak K Antoñana-Vildosola A Valverde P Travecedo MA Jame-Chernaboo Z Schmidt EN. The unique molecular recognition features of Siglec-10: structural insights into sialoglycan and antibody interactions. bioRxiv (2025) 2025.06.10.658867. 10.1101/2025.06.10.658867

[95] Suárez-Álvarez B Fernández-Sánchez A López-Vázquez A Coto E Ortega F López-Larrea C. NKG2D and its ligands: active factors in the outcome of solid organ transplantation? Kidney Int Suppl. (2011) 1:52–7. 10.1038/kisup.2011.13 PMC4089716 25018903

[96] Szczykutowicz J. Ligand recognition by the macrophage galactose-type C-type lectin: self or non-self?—a way to trick the host's immune system. Int J Mol Sci. (2023) 24:17078. 10.3390/ijms242317078 38069400 PMC10707269

[97] Tateno H Ohnishi K Yabe R Hayatsu N Sato T Takeya M. Dual specificity of langerin to sulfated and mannosylated glycans via a single C-type carbohydrate recognition domain *. J Biol Chem. (2010) 285:6390–400. 10.1074/jbc.M109.041863 20026605 PMC2825434

[98] Tector AJ Tector M Copsel S Yu Wang Z Burlak C Reyes LM. Humoral barrier to preclinical and clinical xenotransplantation. Xenotransplantation. (2025) 32:e70056. 10.1111/xen.70056 40492345

[99] Teoh CW Riedl Khursigara M Ortiz-Sandoval CG Park JW Li J Bohorquez-Hernandez A. The loss of glycocalyx integrity impairs complement factor H binding and contributes to cyclosporine-induced endothelial cell injury. Front Med. (2023) 10:891513. 10.3389/fmed.2023.891513 PMC9968885 36860338

[100] Troise D Infante B Mercuri S Lindholm B Kublickiene K Stallone G. Exploring the immunological landscape of ischemia/reperfusion injury and graft rejection in kidney transplantation: shared mechanisms and insights. Cells. (2025) 14:1443. 10.3390/cells14181443 41002408 PMC12468464

[101] van der Zande HJP Nitsche D Schlautmann L Guigas B Burgdorf S. The mannose receptor: from endocytic receptor and biomarker to regulator of (meta)inflammation. Front Immunol. (2021) 12:765034. 10.3389/fimmu.2021.765034 34721436 PMC8551360

[102] van Kooyk Y Ilarregui JM van Vliet SJ. Novel insights into the immunomodulatory role of the dendritic cell and macrophage-expressed C-type lectin MGL. Immunobiology. (2015) 220:185–92. 10.1016/j.imbio.2014.10.002 25454488

[103] van Vliet SJ van Kooyk Y. Sialic acids in cancer biology and immunity—recent advancements. J Biol Chem. (2025) 301:110641. 10.1016/j.jbc.2025.110641 40885395 PMC12494545

[104] Villanueva-Cabello TM Gutiérrez-Valenzuela LD Salinas-Marín R López-Guerrero DV Martínez-Duncker I. Polysialic acid in the immune system. Front Immunol. (2022) 12:823637. 10.3389/fimmu.2021.823637 35222358 PMC8873093

[105] Vrablova V Kosutova N Blsakova A Bertokova A Kasak P Bertok T. Glycosylation in extracellular vesicles: isolation, characterization, composition, analysis and clinical applications. Biotechnol Adv. (2023) 67:108196. 10.1016/j.biotechadv.2023.108196 37307942

[106] Wang A Ribeiro RVP Ali A Brambate E Abdelnour-Berchtold E Michaelsen V. *Ex vivo* enzymatic treatment converts blood type A donor lungs into universal blood type lungs. Sci Transl Med. (2022) 14:eabm7190. 10.1126/scitranslmed.abm7190 35171649

[107] Wang TT Ravetch JV. Functional diversification of IgGs through fc glycosylation. J Clin Invest. (2019) 129:3492–8. 10.1172/JCI130029 31478910 PMC6715372

[108] Wang Y Chen G Pan D Guo H Jiang H Wang J. Pig-to-human kidney xenotransplants using genetically modified minipigs. CR Med. (2024) 5:101744. 10.1016/j.xcrm.2024.101744 PMC11513830 39317190

[109] Wearsch PA Peaper DR Cresswell P. Essential glycan-dependent interactions optimize MHC class I peptide loading. Proc Natl Acad Sci USA. (2011) 108:4950–5. 10.1073/pnas.1102524108 21383180 PMC3064348

[110] West LJ Pollock-Barziv SM Dipchand AI Lee KJ Cardella CJ Benson LN. ABO-incompatible heart transplantation in infants. N Engl J Med. (2001) 344:793–800. 10.1056/NEJM200103153441102 11248154

[111] Wieboldt R Sandholzer M Carlini E Lin C Börsch A Zingg A. Engagement of sialylated glycans with siglec receptors on suppressive myeloid cells inhibits anticancer immunity via CCL2. Cell Mol Immunol. (2024) 21:495–509. 10.1038/s41423-024-01142-0 38448555 PMC11061307

[112] Williams C Pazos R Royo F González E Roura-Ferrer M Martinez A. Assessing the role of surface glycans of extracellular vesicles on cellular uptake. Sci Rep. (2019) 9:11920. 10.1038/s41598-019-48499-1 31417177 PMC6695415

[113] Wu Z Shi J Lamao Q Qiu Y Yang J Liu Y. Glycan shielding enables TCR-sufficient allogeneic CAR-T therapy. Cell. (2025) 188:6317–6334.e21. 10.1016/j.cell.2025.07.046 40845838

[114] Xiao H Woods EC Vukojicic P Bertozzi CR. Precision glycocalyx editing as a strategy for cancer immunotherapy. Proc Natl Acad Sci USA. (2016) 113:10304–9. 10.1073/pnas.1608069113 27551071 PMC5027407

[115] Yokoyama WM Kim S. Licensing of natural killer cells by self-major histocompatibility complex class I. Immunol Rev. (2006) 214:143–54. 10.1111/j.1600-065X.2006.00458.x 17100882

[116] Yue S Wang X Ge W Li J Yang C Zhou Z. Deciphering protein O-GalNAcylation: method development and disease implication. ACS Omega. (2023) 8:19223–36. 10.1021/acsomega.3c01653 37305274 PMC10249083

[117] Zaal A Li RJE Lübbers J Bruijns SCM Kalay H van Kooyk Y. Activation of the C-type lectin MGL by terminal GalNAc ligands reduces the glycolytic activity of human dendritic cells. Front Immunol. (2020) 11:305. 10.3389/fimmu.2020.00305 32161592 PMC7053379

[118] Zeng J Ma M Jiang X Rao Z Huang D Zhang H. Enzymatic conversion of blood group B kidney prevents hyperacute antibody-mediated injuries in ABO-incompatible transplantation. Nat Commun. (2025) 16:1506. 10.1038/s41467-025-56563-w 39929829 PMC11810989

[119] Zeng J Ma M Tao Z Rao Z Wu C Yin S. Enzyme-converted O kidneys allow ABO-incompatible transplantation without hyperacute rejection in a human decedent model. Nat. Biomed. Eng. (2025):1–17. 10.1038/s41551-025-01513-6 41044341

[120] Zeng S Wen Y Yu C. Desialylation of ATG5 by sialidase (NEU1) promotes macrophages autophagy and exacerbates inflammation under hypoxia. Cell Signal. (2023) 112:110927. 10.1016/j.cellsig.2023.110927 37844713

[121] Zhao D Abou-Daya KI Dai H Oberbarnscheidt MH Li XC Lakkis FG. Innate allorecognition and memory in transplantation. Front Immunol. (2020) 11:918. 10.3389/fimmu.2020.00918 32547540 PMC7270276

[122] Zheng Y Ma X Su D Zhang Y Yu L Jiang F. The roles of Siglec7 and Siglec9 on natural killer cells in virus infection and tumour progression. J Immunol Res. (2020) 2020:6243819. 10.1155/2020/6243819 32322597 PMC7165337

[123] Zhu W Zhou Y Guo L Feng S. Biological function of sialic acid and sialylation in human health and disease. Cell Death Discov. (2024) 10:415. 10.1038/s41420-024-02180-3 39349440 PMC11442784

